# Intensive Postural and Motor Activity Program Reduces Scoliosis Progression in People with Rett Syndrome

**DOI:** 10.3390/jcm11030559

**Published:** 2022-01-22

**Authors:** Alberto Romano, Elena Ippolito, Camilla Risoli, Edoardo Malerba, Martina Favetta, Andrea Sancesario, Meir Lotan, Daniel Sender Moran

**Affiliations:** 1 Department of Health System Management, Ariel University, Ariel 4070000, Israel; danielm@ariel.ac.il; 2 Movement Analysis and Robotics Laboratory, Intensive Neurorehabilitation and Robotics Department, Bambino Gesù Children’s Hospital, 00165 Rome, Italy; martina.favetta@opbg.net (M.F.); andrea.sancesario@opbg.net (A.S.); 3 Centro AIRETT Ricerca e Innovazione (CARI), Research and Innovation AIRETT Center, 37122 Verona, Italy; 4 SMART Learning Center, 20133 Milan, Italy; elena.ippolito@centrosmart.it; 5 Department of Radiological Functions, Radiology Unit, Guglielmo da Saliceto Hospital, 29121 Piacenza, Italy; camilla.risoli11@gmail.com; 6 Poliambulatorio Health Medical, 29122 Piacenza, Italy; edoardo.malerba11@gmail.com; 7 Department of Physiotherapy, Ariel University, Ariel 4070000, Israel; meirlo@ariel.ac.il; 8 Israeli Rett Syndrome National Evaluation Team, Ramat Gan 5200100, Israel

**Keywords:** Rett syndrome, scoliosis, motor skills, telerehabilitation, physical therapy modalities, home exercise program

## Abstract

Background: A scoliosis prevalence of 94% was reported in the population with Rett syndrome (RTT), with an annual progression rate of 14 to 21° Cobb which may result in pain, loss of sitting balance, deterioration of motor skills, and lung disfunction. This paper describes the efficacy of an intensive conservative individualized physical and postural activity program in preventing scoliosis curvature progression in patients with RTT. Methods: Twenty subjects diagnosed with RTT and scoliosis were recruited, and an individualized intensive daily physical activity program was developed for each participant. Each program was conducted for six months by participants’ primary caregivers in their daily living environment. Fortnightly remote supervision of the program implementation was provided by an expert therapist. Pre- and post-intervention radiographs and motor functioning were analyzed. Results: An averaged progression of +1.7° ± 8.7° Cobb, over one year (12.3 ± 3.5 months) was observed in our group, together with motor function improvements. A relation between curve progression and motor skill improvement was observed. Conclusions: The intervention prevented scoliosis progression in our group. The achievement of functional motor improvements could enable better body segment control and muscle balancing, with a protective effect on scoliosis progression. The intervention was effective for individuals with RTT across various ages and severity levels. Individual characteristics of each participant and the details of their activity program are described.

## 1. Introduction

Rett syndrome (RTT) is a severe neurodevelopmental disease caused by a mutation in the MECP2 gene located on the X chromosome [1], affecting about 1/10,000 females worldwide [2,3,4]. Scoliosis is the most common orthopedic comorbidity in RTT [5,6,7], showing a prevalence of up to 94% [7,8,9,10,11]. Reported median age of scoliosis onset in RTT is at 9.8 years, with 25% of subjects affected by the age of 6 years, about 79% by the age of 13 years, and 85% by the age of 16 years or older [7,9,12,13]. Scoliosis in RTT is neurogenic in origin and can result in pain, loss of sitting balance, deterioration of motor skills, and progressive restrictive lung disease development [14]. Diminished early development, inability to sit and walk without support, the onset of puberty, and enhanced clinical severity are also associated with developing scoliosis at an early age [11,13,15].

The case literature suggests a fast mean curve progression in this population, ranging from 14 to 21° Cobb per year, accompanied by an acceleration in the progression at puberty with an average age of 13 years [11,14,16]. Moreover, in a large cohort study analyzing radiographs of 128 girls and women with RTT followed for two years (three radiographs for each subject), the magnitude of scoliosis was found to generally increase with age with a yearly 0.438-unit increase in the square root of the baseline Cobb’s angle (95% confidence interval 0.374–0.503) [17].

A corset is usually suggested to individuals with RTT with Cobb’s angle greater than 20–25°, and spinal fusion is considered if Cobb’s angle progresses over 40–50° [12,15,18].

Spinal surgery is considered the definitive management of neuromuscular scoliosis [19]. In most cases, posterior-only spinal fusion is performed [20]. However, if an anteroposterior surgery is required, a single-stage approach is preferable to reduce anesthetic and surgical complications [12]. Fixation to the pelvis is indicated in non-ambulant children with pelvic obliquity [12] and in the presence of a preceding rapid decline in neurological function with a lethargic presentation and uncontrolled, treatment-resistant daily seizures [21]. Surgery should not be delayed until skeletal maturity has been achieved. Barney and colleagues suggested the need for RTT-specific post-operative pain management [22]. The authors reported that, after spinal fusion, patients with RTT received fewer total doses of opioids compared to girls with cerebral palsy and idiopathic scoliosis. With modern technology, severe curves can be safely treated [21] and positive outcomes following spinal surgery were reported [23,24,25,26]. Even so, the decision to perform the operation is challenging for the family due to anxiety about their daughter undergoing such a complex procedure [8,24]. Moreover, a large cohort study reported that 18.5% of subjects with RTT and scoliosis had spinal instrumentation [15], showing that the vast majority of this group of clients will not undergo surgery and therefore will rely on non-surgical, conservative treatment. Therefore, the development and evaluation of such programs are critical for individuals with RTT.

Available conservative interventions for scoliosis include observation and monitoring, bracing, orthotics, and physical therapy [27,28,29]. As specific guidelines for the management of scoliosis for people with RTT were published [12], the available treatments for scoliosis were here described in the light of such guidelines. The scoliosis monitoring for these patients should start before the diagnosis of scoliosis. Before-diagnosis monitoring strategies for people with RTT include genetic testing, parental teaching on the characteristic aspects of scoliosis, and physical assessment upon the diagnosis of RTT and at least every six months after that [12]. After diagnosing scoliosis, yearly spine radiographs are suggested to monitor the curve progression. The collection of radiographs every six months is recommended if the Cobb’s angle progresses over 25° before the skeletal maturity. The maintenance of physical examinations every six months is advised after the scoliosis diagnosis [12]. Bracing strategies were suggested in the literature for neuromuscular scoliosis management among its conservative treatments. A recent literature review identified 23 typologies of braces proposed to care for scoliosis curves of different types (“C” or “S” shaped), levels (thoracic, lumbar, and thoracolumbar), and entities, using different approaches (passive and active correction) [30]. Indications for bracing are stronger in younger children and more controversial in adolescents. Bracing aims to contain the curve until skeletal maturity [31] and regain the sitting position [32]. However, the role of bracing in patients with neuromuscular scoliosis is contentious and there is no compelling evidence supporting the use of braces to prevent the deformity progression in neuromuscular scoliosis [19,32]. There is no consensus among experts on RTT that bracing is beneficial in reducing the scoliosis progression in this population [12]. Nevertheless, bracing may be found valuable if support is needed in a sitting position (usually for very hypotonic children who find it difficult to maintain an erect posture for long periods) or to delay the necessity of surgery [12,33,34]. 

When considering conventional interventions regarding scoliosis, there is a consensus among experts that efforts should be made to maintain the ability to walk and increase duration and distance walked to counteract the curve progression [19,27,35]. However, the capacity of physical therapy treatments to reduce scoliosis or prevent mild scoliosis from rapidly progressing has, to date, received mild consideration in the literature [35]. Although, so far, physical therapy interventions have not been found to improve etiological factors for neuromuscular scoliosis or prevent progression of established scoliosis, they were reported as helpful in avoiding any adverse effects, such as prolonged brace use, prevention or prolonging onset of joint contractures, and maintenance of both chest mobility and respiratory excursion [27,32]. The implementation of physical therapy for scoliosis must be based on the particular spinal deformity characteristics and requires specifically trained therapists and clinicians [28].

To our knowledge, only one anti-scoliotic physical therapy intervention explicitly directed to patients with RTT was noted in the literature [35]. This is a specific physical therapy regimen comprised of an intensive program of postural positioning and enhanced activity level carried out in joint cooperation with the participant’s family members and caregivers (within the educational facility). The intervention was conducted with a 5-year-old girl with RTT whose Cobb’s angle reduced from 30 to 20° over an 18-month treatment period, through the use of positioning equipment, postural strategies (activating automatic equilibrium reactions, which oppose the natural scoliotic curve), and a motor activity program (walking or standing) during waking hours. Similar programs were found helpful to increase the motor function of five girls with RTT in Ireland [36], and 17 girls and women with RTT in Italy [37], providing evidence of feasibility and acceptability of this treatment in a broader age and functional range of participants. Yet, to date, no anti-scoliotic, conventional, physical regime was evaluated for a cohort of participants with Rett syndrome.

The present paper aims to describe the efficacy of such a physical activity program in preventing the scoliosis curvature progression in a group of patients with RTT and analyzing the variables that can affect the intervention efficacy. The proposed intervention is ecological and intensive; it was carried out by primary caregivers within the participants’ daily living environment and was planned and remotely supervised fortnightly by therapists with expertise in carrying out motor rehabilitation treatments for people with RTT. The proposed intervention was developed based on the one proposed by Lotan and colleagues [35].

## 2. Materials and Methods

### 2.1. Study Design

A single-subject A-B-A design was applied.

The dependent variable was the Cobb’s angle changes before (T1) and after (T2) the intervention, 12.3 ± 3.5 months apart.

The independent variable was the implementation of a six-month individualized motor and postural activity program carried out by participants’ parents and care providers within the participants’ daily living environment for one hour a day, five days a week.

### 2.2. Participants

Twenty Italian girls and women with genetically confirmed classic RTT and diagnoses of scoliosis participated in this research. Participants were recruited from the Italian Rett Association (AIRett) database. All participants lived at home with their parents. One subject was waiting for scoliosis surgery at enrolment (T1). Descriptive statistics of participants’ age, RTT severity level, and daily physical activity level are presented in Table 1.

### 2.3. Outcome Measures

#### 2.3.1. RTT Severity Level

The Rett Assessment Rating Scale (RARS) was used to assess the severity of clinical manifestation of RTT [38]. The RARS was established by a standardization procedure, proving that the instrument is statistically valid and reliable [39].

#### 2.3.2. Activity Level

Participants’ parents completed the modified Bouchard activity record (m-BAR) to evaluate their daughter’s physical activity level [40,41]. Previous studies demonstrated good reliability of the m-BAR as a daily activity level measure for individuals with RTT [40,41]. The m-BAR was collected at T1 to help the researchers figure out how active participants were during their typical day.

#### 2.3.3. Motor Functioning

The motor functioning level was scored at T1 and T2 using the Rett Syndrome Motor Evaluation Scale (RESMES) [39,42]. This RTT-specific gross motor evaluation scale showed optimal inter-rater agreement among clinicians and strong internal consistency [42].

#### 2.3.4. Scoliosis Severity

A clinical score for the scoliosis severity was given to each participant at T1 to help understand the curve’s characteristics. The scoliosis severity level was coded with a 0 to 3 ordinal score (0: no scoliosis; 1: mild scoliosis, curve visible only on thorough examination in forward bending; 2: moderate scoliosis, obvious curve in both upright and forward bending; 3: severe scoliosis, pronounced curve preventing upright position without external support) as suggested for individuals with RTT by Rodocanachi and colleagues [43].

**Figure 1:**
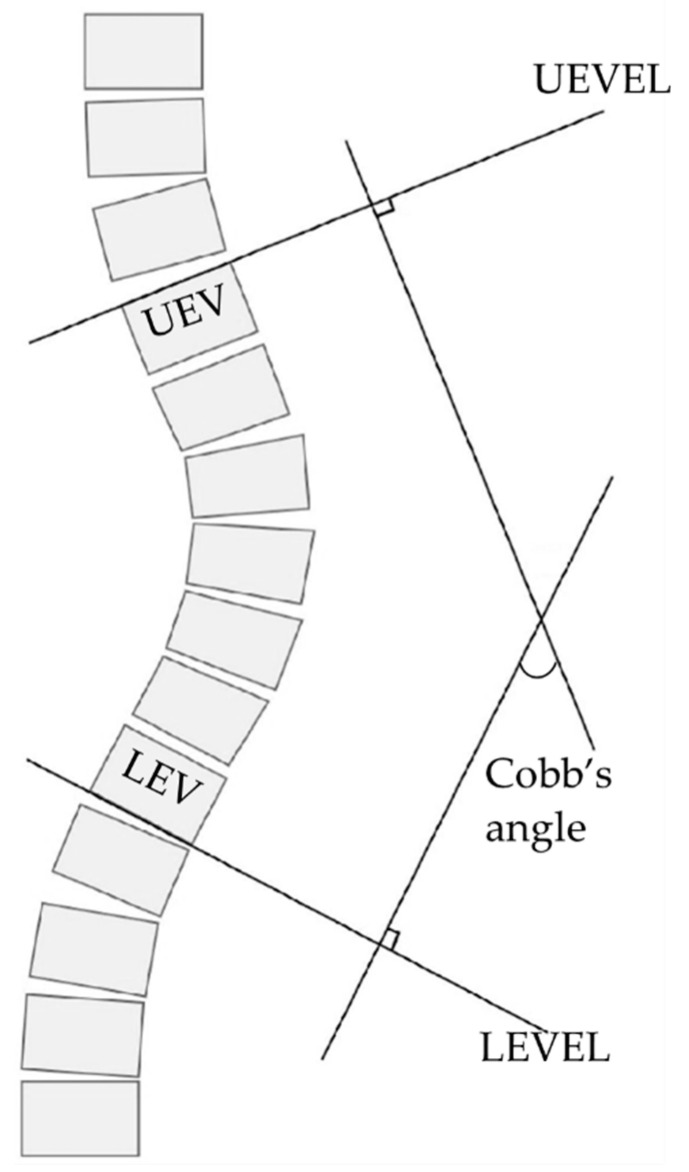
Visual explanation of the method used to measure the Cobb’s angle. Upper and lower end vertebra endplate lines (UEVEL; LEVEL).

Participants’ coronal Cobb’s angles were digitally measured by two independent assessors through manual use of the RadiAnt DICOM Viewer software (version 2020.2.1—Medixant, Poznan, Poland) “Cobb’s angle” tool on the participants’ anterior–posterior radiograph images. The Cobb angle is a standard quantification value of scoliosis certified by the Scoliosis Research Society. This study has adopted it as an objective measure, to quantify state of scoliosis at T1 and change in scoliosis, based on the radiograph images [44]. The manual measurement of Cobb’s angle required determining the upper/lower end vertebras (UEV/LEV) on the participants’ whole spine antero–posterior radiograph images and drawing two lines corresponding to the upper and lower end vertebra endplate lines (UEVEL; LEVEL). Then, two lines respectively perpendicular to the UEVEL and LEVEL are drawn. The angle included between the two perpendicular lines represents the Cobb’s angle. Visual explanation of Cobb’s angle measurement procedure is available in Figure 1. Each participant’s radiographs were collected at T1 and T2 at the same facility, in the same posture, and by the same technician following international guidelines [12]. If a double curve was present, the bigger curvature was considered.

Detailed clinical descriptions of motor functioning changes between T1 and T2 due to the present intervention program will be presented in a future article.

### 2.4. Procedure

The present research was conducted following the Declaration of Helsinki principles and approved by the Ariel University Institutional Review Board (AU-HEA-ML-20190326-1). Before starting the intervention, informed consent for participation and study publication was collected for all participants from their legal guardians. Each participant was then clinically evaluated at her home by two therapists experienced in treating people with RTT (pre-intervention evaluation meeting—T1). At T1, the levels of RTT severity, daily physical activity, motor functioning, and scoliosis severity were assessed, and relevant information related to participants’ clinical conditions (such as the presence of comorbidities typically associated with RTT), daily activities, routines, and living environments were collected. Participants’ parents and referred rehabilitation professionals (when available) participated in this meeting.

At the end of the T1 meeting, a draft of individualized motor activities and the postural program was prepared relying on the collected information. The draft was then discussed with participants’ parents and referred therapists until consensus on activity characteristics and their execution timetable was reached. Each program included 4–7 (median: 5) therapeutic activities that parents could perform at home and that could be easily integrated into the participant’s daily routine. Those activities were based on passive (hypercorrective) [35,45] and active (causing the participant to use balance reactions while sitting, standing, and walking, working asymmetrically and harder with the extensors and side flexors of the convexity side of her body, against the natural pull of scoliosis) [35,46] postural strategies and on the performance of motor tasks that strengthened the trunk muscles. A full description of each activity included in each program is available as Supplementary Material (see “Text file—Supplementary material”, which illustrates each participant’s activities carried out during the intervention). Each activity in the program was shown and taught to the participants’ parents, other involved caregivers (e.g., babysitters and teachers), and therapists in vivo during the first evaluation meeting where possible, and through video call and digital material sharing (such as figures and videos) in the other cases. Each participant was required to perform the programmed activities for at least one non-consecutive hour per day, five days a week, for six months. The progress of each program was supervised fortnightly for the first three months of the intervention by one of the therapists who had created the programs via video call with the involved caregivers. The supervision meetings lasted for one hour each and were aimed to support the program execution, answering caregivers’ questions, adapting the program to the emerged needs, solving problems, rearranging timetables, adapting the suggested exercises, and, if needed, setting new activities. At the end of the intervention, participants’ motor functioning level was assessed again, and the post-intervention spine radiographs were collected (T2).

### 2.5. Statistical Analysis

As the obtained results were not normally distributed, non-parametric statistics were used for the data analysis. Wilcoxon signed-rank test was conducted to compare the participants’ Cobb’s angles and RESMES total scores collected at T1 and T2. If a statistical difference was found within the above-reported comparisons, the size of the effect was calculated using the matched-pairs rank-biserial correlation [47,48]. The effect size can be defined as the degree to which a null hypothesis is false or the degree to which the phenomenon is present in the population [49]. Cohen [50] proposed widely used interpretative guidelines for the effect size, considering it small if between 0.200 and 0.500, medium between 0.500 and 0.800, and large if above 0.800. However, a meaningful interpretation of the magnitude of a treatment effect requires a comparison of the result to a frame of reference, such as previous findings [51]. Accordingly, Cohen suggested that his interpretative framework should only be used when the discipline lacks specific guidelines [50,52]. Therefore, for the present study, the effect size interpretation followed the empirically derived guidelines proposed by Kinney and colleagues [53] for rehabilitation studies. Accordingly, the effect size was considered small if between 0.140 and 0.310, medium if between 0.310 and 0.610, and large if above 0.610 [53].

Relations between Cobb’s angles changes with participant’s age, Cobb’s angles at T1, level of RTT severity, daily physical activity at T1, motor function at T1, and the variation of motor functional levels that occurred between T1 and T2 were explored using Spearman’s rank correlation coefficient. This non-parametric test assesses how well a monotonic function can describe the relationship between two variables and is equal to using the Pearson correlation between the rank values of two variables. The threshold for significance for the comparisons above has been assumed as α = 0.05. No correction for multiple comparisons was applied [54].

## 3. Results

All our participants carried out their individualized program for its entire duration (six months). The descriptive statistics of Cobb’s angles measured at T1 and T2 and the difference between Cobb’s angles measured at the two evaluations are collected in Table 2.

**Figure 2:**
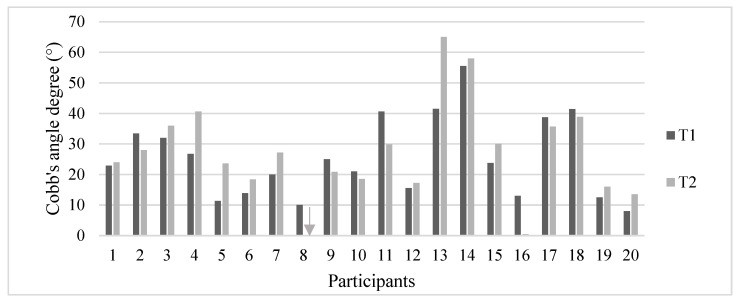
Cobb’s angles of each participant at T1 and T2. Gray arrow (participant no. 8) represents the absence of scoliosis at T2.

Figure 2 shows Cobb’s angles of each participant at T1 and T2. Four of the eight participants (50%) who reduced their Cobb’s angle improved their curve for at least five degrees. On the other hand, a worsening of more than 5° Cobb was observed in 6 out of 12 subjects (50%) who worsened their curves. Considering as a curve progression a Cobb’s angle increment of more than 5°, the scoliosis of 70% of participants did not deteriorate at T2.

The Wilcoxon signed-rank test showed no statistical difference between Cobb’s angles collected at T1 and T2 (*p*= 0.400). Descriptive statistics of RESMES total and subscales results collected at T1 and T2, and corresponding *p*-values and size effects, are collected in Table 3. On average, the motor function of participants in our group improved at the end of the intervention. Statistically significant improvements were found for the transitions and stair-climbing RESMES subscales but without a size effect. On the other hand, the total RESMES score showed a statistically significant reduction after the intervention (representing an improvement in global motor functioning) with a large size effect.

A moderate positive correlation (*p* = 0.008; ρ = 0.574) was found between the Cobb’s angles variation and functional motor level variation (evaluated with RESMES) between T1 and T2. No other correlations were found between the Cobb’s angle changes at T2 and the other investigated variables. Participants’ individual data are available as Supplementary Material (see Tables S1–S3 in “Tables—Supplementary material”, which illustrates each participant’s age, genetic mutation, RARS scores, daily physical activity level, scoliosis severity, Cobb’s angles, and RESMES scores measured at T1 and T2).

## 4. Discussion

This paper presented the effect of a six-month remotely supervised individualized motor and postural activity program carried out by parents, therapists, and primary caregivers (when available) with their daughters and clients with RTT. The results suggest that the proposed intervention effectively prevented the participants’ scoliosis progression as 70% of participants did not worsen their curves. The available literature reported the absence of progression in 0–17% of cases. [11,16,55] One study reported that 62% of the 21 included participants (age range= 4.3–18 years) did not worsen their curve [56], but this result was never replicated. This finding is significant for the population with RTT that shows a high prevalence of scoliosis (up to 94%). According to a large observational study, only 18.5% of people with RTT and scoliosis undergo spinal surgery [15]. Therefore, most subjects with RTT rely on non-surgical, conservative intervention, such as the one suggested within the current intervention program, to cope with the scoliosis progression. As of today, the existing literature regarding conservative treatments for scoliosis is limited, and the current article presents the first evidence on the established efficacy of a conservative intervention coping with scoliosis progression in people with RTT. The case series available in the literature reported an average annual curve progression ranging from 14–21° Cobb with an acceleration in the progression during adolescence [11,14,16]. Compared to them, participants in our group have shown a much lower progression rate (average Cobb’s angle change= +1.7° ± 8.7° in 12.3 ± 3.5 months) even though the average age of the current intervention program’s participants was mid-puberty (15.6 years). Moreover, the participants’ motor functional level improved at T2 with a large effect size when considering the global motor function. As the intervention was focused on daily physical activities, this result suggests that the daily performance of such activities had a positive effect also on the participants’ global motor functioning. Furthermore, even though no statistical differences or significant effect size were found, a positive trend was identifiable in all the RESMES subscales (except the “running” subscale). The findings of the current intervention program agree with previous reports, suggesting an improvement in functional abilities in RTT after two months of a daily treadmill training program [57]. Similar programs (remote supervision of intensive activity programs for individuals with RTT) performed in different groups and at different locations (Ireland and Italy) have also presented similar results [36,37,58,59]. The correlation between Cobb’s angles and RESMES total score variations suggests that a greater improvement in motor functioning is related to a more significant benefit to the scoliotic curve. These findings can be explained by the fact that higher-level functional abilities necessitated more complex dynamical control of the body that requires better integration of sensory information and increased core muscle strength and usage, leading to a better balance in back muscle strength. In support of this consideration, our group statistically improved stair climbing ability, which was related in previous research to better scoliosis outcomes in this population [60]. Moreover, these considerations echo a previous report of Bisgaard and colleagues describing a negative relation between scoliosis entity and motor functioning in adults with RTT [61]. The authors highlighted the need for lifelong rehabilitation and promotion of an active lifestyle to maintain physical health and functional level, which the authors of the current article wholeheartedly support.

**Figure 3:**
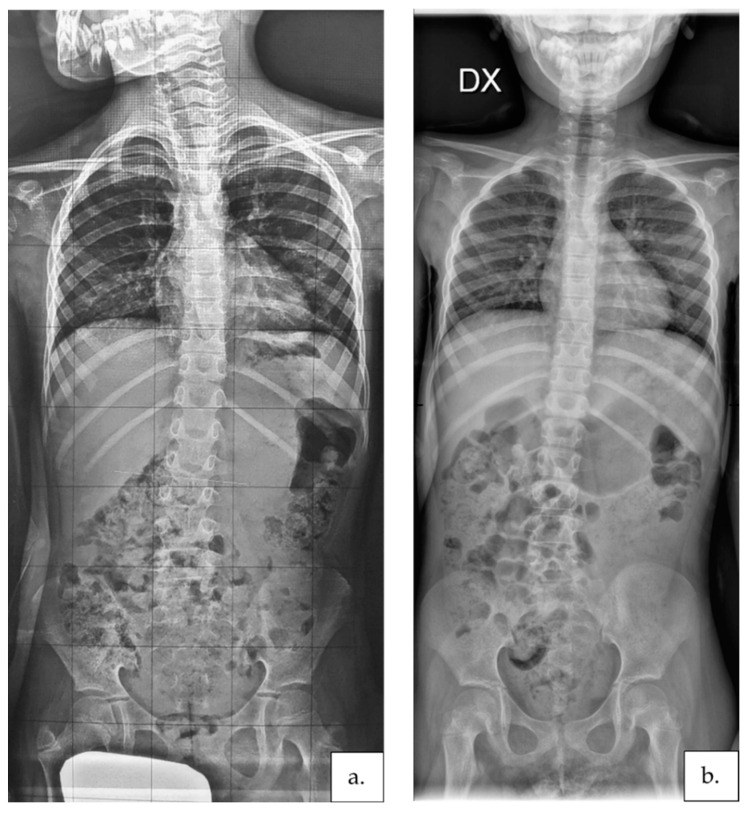
Participant no. 8′s X-rays collected before (**a**) and after (**b**) the intervention. She was 6.5 years old at T1. At baseline, she could walk for more than 10 steps supported by one hand and independently stand for 30 s. Lack of efficient balance reaction was recognized at T1. Therefore, her program included intensive balance training and asymmetrical posture maintenance. At the end of the intervention, she could walk for more than 10 steps and stand for more than one minute independently. An improvement of 10° Cobb was recognized at T2.

Furthermore, when looking at the individual results, in two cases (participants no. 8 and 16) showing unstructured flexible scoliosis, the intervention was able to eliminate the scoliotic curve completely. This result is highly significant as no spontaneous scoliosis curve regression was ever reported in people with RTT. These participants were the youngest in our group and the only two who also learned to walk independently during the intervention. Walking ability development, maintenance, and promotion for as long as possible are strongly recommended by the guidelines for scoliosis management in RTT [12] and were found to be associated with a reduced risk of severe scoliosis development in this population [15,62]. Participant no. 8′s case description and pre- and post-intervention radiographs are available in Figure 3.

On the other hand, the curve of two participants (no. 4 and 13) progressed for at least 14° despite the intervention. In both cases, between T1 and T2, the researchers recognized a progressive reduction in mobility. In one case (participant no. 4), this reduction was driven by the scoliosis progression that made it harder and harder for her to move. In the other case (participant no. 13), the increased immobility could be attributed to progressive and painful supination of the right foot that prevented her from standing and walking for a long duration. Therefore, the authors would like to reiterate the importance of starting the intervention at an early age and of the continuative promotion of the motor skills (walking in particular) of people with RTT, as was repeatedly suggested by experts in the field of RTT [63,64].

**Figure 4:**
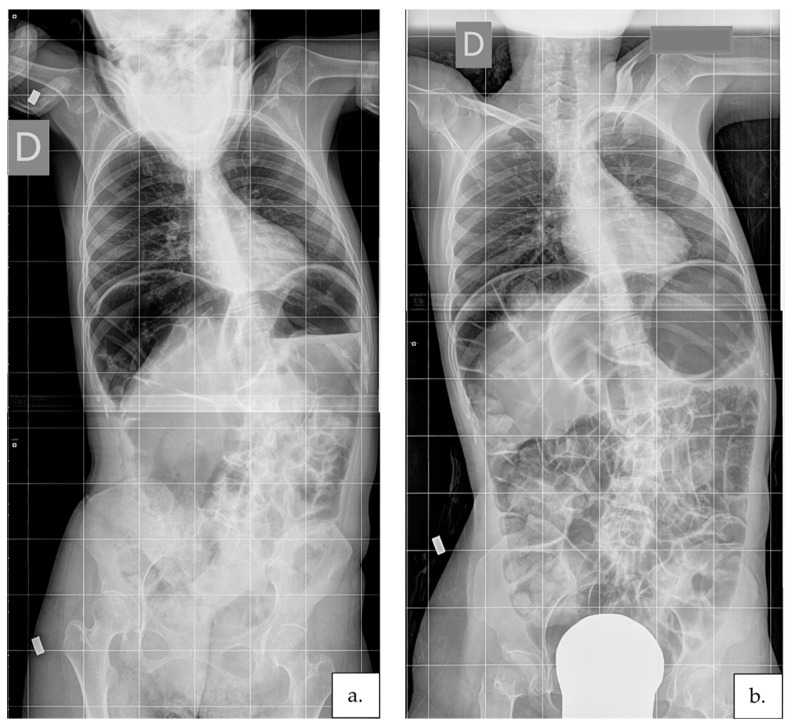
Participant no. 11′s radiographs collected before (**a**) and after (**b**) the intervention. She was 31.6 years old at T1 and used to sit with trunk supported for most of her waking hours. She was able to stand and walk only with significant support given at the trunk from behind. During the intervention, she started a daily program of unsupported asymmetrical sitting, walking on a treadmill, and overcorrecting passive posture maintenance. At the end of the intervention, she was able to walk more than 10 steps and stand for more than one minute with support given to the distal section of both hands. An improvement of 11° Cobb was recognized at T2.

The absence of correlations between Cobb’s angle variations and the other investigated variables, together with the wide range of participants’ age and RTT severity level, suggests that the proposed intervention can effectively limit the scoliosis progression of people with RTT at any age and severity level. Interestingly, among the four participants older than 20 years in our group (no. 2, 3, 11, and 18), three reduced their curve (in one case, by 11°—subject no. 11—Figure 4).

Moreover, the only subject showing a severe manifestation of RTT (RARS score >81—subject no. 17) also improved her curve of three degrees Cobb (showing the absence of progression). She used to lie down or be fully supported throughout her waking hours. Therefore, in her case and others, the activity program led to a considerable increment in her daily abilities (standing and walking activity with support), bringing positive results for her scoliosis. The above presented cases, as well as others presented within the scope of the present article, reiterate the fact that physical activity is extremely important to those with RTT continuously from a young age and that improvements in functional abilities can be achieved by this group of clients at all severity levels and at all ages.

The main limitation of the present research is that the study design limits the generalization of the results. A randomized controlled trial with a more solid baseline and follow-up is required to verify the robustness of the results. Moreover, the small sample size and the absence of a control group limit the presented results’ external validity and robustness. Future studies with larger samples and control groups are needed to establish the cause/effect relationship between the proposed intervention and the obtained results and compare the intervention benefit with other treatment options (such as standard care or bracing regimens). However, the presented investigation demonstrated the feasibility of this type of intervention, proving its effectiveness, in a group of 20 participants with RTT. Another limitation relates to the lack of kyphosis and pelvic obliquity data collection. This information could provide more insight into the participants’ full clinical pictures and highlight possible treatment effects on such conditions. Finally, even though one participant qualified as a surgery candidate, the absence of data on the pre-intervention progression trajectory of scoliosis development impeded the evaluation of the proposed treatment efficacy in preventing the surgical intervention.

## 5. Conclusions

The present article proposes an effective intervention to prevent scoliosis progression of people with RTT, a population with no established conservative treatments for spinal asymmetries until now. The proposed program was found to be feasible for people with RTT across a wide range of ages and severity levels and can represent a valid strategy to prevent or delay the need for a complex surgery such as spinal fusion. A detailed description of each participant’s characteristics and the therapeutic program was presented and can be found online as Supplementary Materials.

## Figures and Tables

**Table 1:** Descriptive statistics of participants’ ages, RARS total and subscale scores, and m-BAR scores.

		Mean (SD)	Median	Range
Age		15.6 (8.4)	13.2	3.8–38.3
RARS	Cognitive	13.4 (2.8)	13.5	9–18.5
	Sensory	3.2 (1.1)	3.0	2–5.5
	Motor	10.4 (2.1)	10.3	5.5–13
	Emotional	3.2 (0.8)	3.0	2–5
	Independence	10.7 (1.8)	11.3	4.5–12
	Rett characteristics	23.8 (4.5)	24.3	15–33
	Total	64.7 (9.5)	66.0	45.5–81.5
m-BAR		92.8 (26.1)	93.5	52–145

Abbreviation list: SD = Standard Deviation; RARS = Rett Assessment Rating Scale; m-BAR = modified Bouchard activity record.

**Table 2:** Descriptive statistics of participants’ Cobb’s angles at T1 and T2 divided between those whose scoliosis worsened, those whose curve improved, and all participants together. Delta (∆) Cobb’s angles represent the simple difference between Cobb’s angle measured at T2 and T1. Positive values represent a worsening, while negative values represent an improvement in the scoliotic curve.

		Worsening	Improving	All
No. (%)		12 (60%)	8 (40%)	20 (100%)
Cobb’s angles T1 (°)	Mean (SD)	23.6 (13.8)	27.9 (12.5)	25.3 (13.2)
	Median	21.5	29.2	23.3
	Range	8–56	10–41	8–56
Cobb’s angles T2 (°)	Mean (SD)	30.8 (16.5)	21.5 (14.8)	27.1 (16.1)
	Median	25.6	24.4	25.6
	Range	14–65	0–39	0–65
∆ Cobb’s angles (°)	Mean (SD)	7.2 (6.5)	−6.4 (4.1)	1.7 (8.7)
	Median	5.0	-4.8	2.1
	Range	1–23	−13–-2.5	−13–23

Abbreviation list: SD = Standard Deviation; T1 = pre-intervention evaluation meeting; T2 = post-intervention evaluation meeting.

**Table 3:** Descriptive statistics and corresponding *p*-value and effect size of participants’ RESMES total and subscales scores.

			Mean (SD)	Median	Range	*p*-Value	Effect Size
RESMES Subscales	Standing	T1	3.2 (4)	1	0–11	0.098	\
		T2	2.6 (3.5)	0	0–10		
	Sitting	T1	1 (2.3)	0	0–9	0.066	\
		T2	0.4 (1)	0	0–3		
	Transition	T1	14.6 (6.7)	16	0–24	0.034 *	0.138
		T2	14.1 (6.7)	13.5	0–24		
	Walking	T1	7.6 (7)	5.5	0–18	0.094	\
		T2	7.1 (6.7)	4.5	0–18		
	Running	T1	4 (0)	4	4–4	1.000	\
		T2	4 (0)	4	4–4		
	Stairs	T1	6.5 (1.8)	6.5	2–8	0.038 *	0.071
		T2	6.1 (1.9)	6	2–8		
RESMES Total		T1	36.8 (19.3)	32.5	6–70	<0.001 *	0.648
		T2	34.3 (18.1)	28.5	6–66		

Abbreviation list: SD = Standard Deviation; RESMES = Rett Syndrome Motor Evaluation Scale; T1 = pre-intervention evaluation meeting; T2 = post-intervention evaluation meeting. *: *p* < 0.05.

## Data Availability

Data available as Supplementary Material (see “Tables—Supplementary material”).

## Supplementary Materials

The following are available online at https://www.mdpi.com/article/10.3390/jcm11030559/s1, Text file S1: List of proposed activities for each participant, Tables—Supplementary material: Participant’s age, genetic mutation, RARS scores, daily physical activity level, scoliosis severity, Cobb’s angles, and RESMES scores measured at T1 and T2.
