# Supplementary material for: Intensive Postural and Motor Activity Program Reduces Scoliosis Progression in People with Rett Syndrome
Source: J Clin Med. 2022 Jan 22;11(3):559. doi: 10.3390/jcm11030559 (PMC8837095; doi:10.3390/jcm11030559)
Supplement: Supplementary file 1 [file jcm-11-00559-s001.zip › File S1.pdf]

List of proposed activities for each participant

Summary:

Participant no. 1 .... pag. 2
Participant no. 2 .... pag. 3
Participant no. 3 .... pag. 5
Participant no. 4 .... pag. 6
Participant no. 5 .... pag. 7
Participant no. 6 .... pag. 9
Participant no. 7 .... pag. 11
Participant no. 8 .... pag. 13
Participant no. 9 .... pag. 15
Participant no. 10 .... pag. 16
Participant no. 11 .... pag. 18
Participant no. 12 .... pag. 20
Participant no. 13 .... pag. 22
Participant no. 14 .... pag. 24
Participant no. 15 .... pag. 26
Participant no. 16 .... pag. 28
Participant no. 17 .... pag. 30
Participant no. 18 .... pag. 31
Participant no. 19 .... pag. 33
Participant no. 20 .... pag. 35

Participant no. 1

1. To stretch the muscles of the left side of the trunk each time she is sitting in her chair, she should sit with an elevation under her right buttock and right leg (e.g., a folded towel) while keeping her left elbow propped up on a high pillow (see Figure 1).

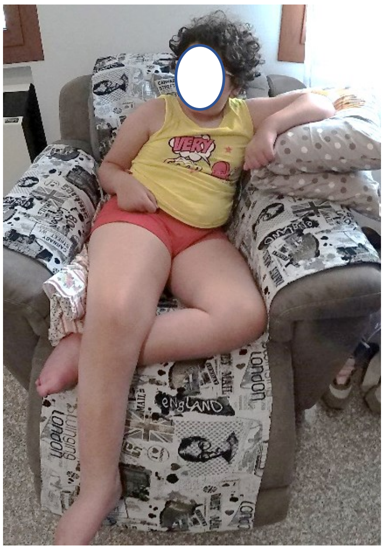

Figure 1

2. To achieve passive correction of scoliosis during sleep, she should sleep inside a "U" shaped pillow placed under the apex of the scoliotic curve, under her right side, to extend the left side of the trunk (see Figure 2).

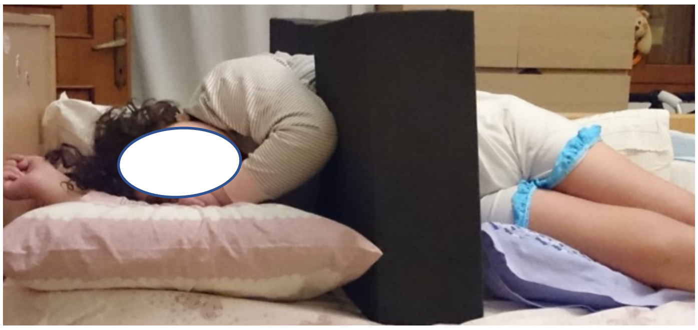

Figure 2

3. Have her stand inside the standing frame by wedging a pillow against the right side of her trunk at the apex of the scoliotic curve. Have her keep her left elbow tucked into the chest support in front of her (see Figure 3a,b). Have her stand inside the standing frame at least 30 consecutive minutes per session (if you can leave her for even longer, even better).

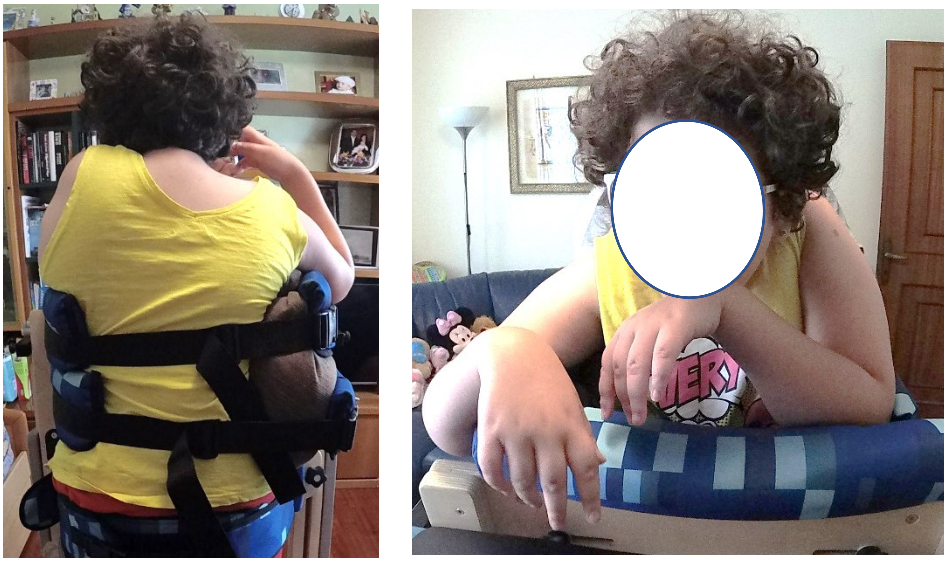

Figure 3a,b

4. Have her sit independently on the arm of her chair with her feet supported and slightly unbalanced towards her left (see Figure 4). Initially, she will need your help to find balance. Once she has found her balance, try letting her sit independently while watching her favorite cartoon on TV. Have her sit in this way initially for 3/5 minutes per session and gradually increase the exercise duration to reach 10 minutes.

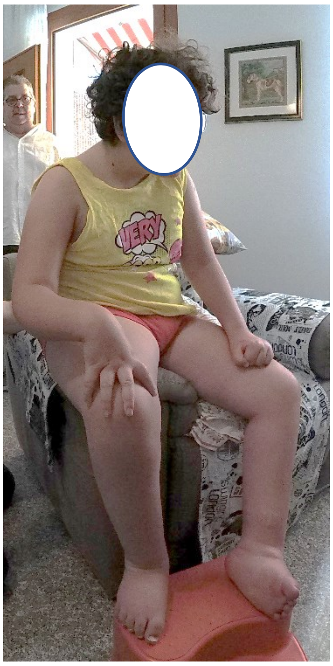

Figure 4

5. Have her walk for small distances in the house at home and school, holding her by the hands. To increase her physical activity, try to get her to do at least four walks more than she currently does.

6. Have her stand leaning with her arms on her lifter's canvas support. The lifter should be held at full height, and she should be slightly leaning forward so that she does not risk falling backward (see Figure 5). Have her stand like this every day, initially for 3/5 minutes per session, and gradually increase the exercise duration to reach 10 minutes.

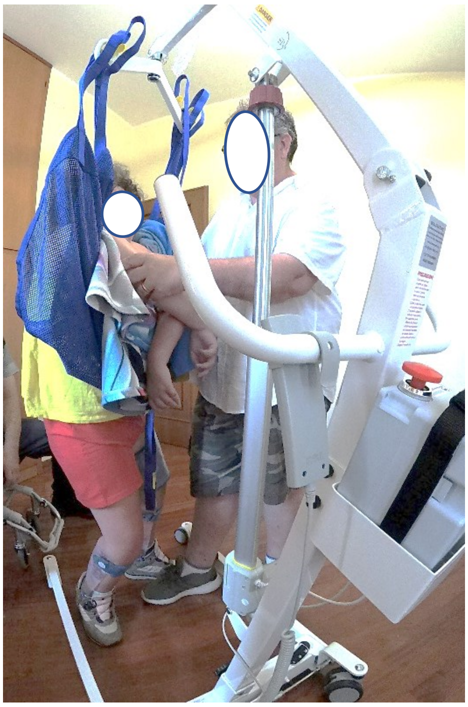

Figure 5

Participant no. 2

1. Whenever she has to enter or leave the house, have her go up and down the stairs as much autonomously as possible. Stand in front of her to both go up and down. To go up, help her hold onto the handrail with one hand and hold her other wrist with the other hand. To go down, help her hold onto the handrail with one hand and let her lean on your shoulder with the other hand. Use your free hand to help her keep her balance (see Figure 6a,b).

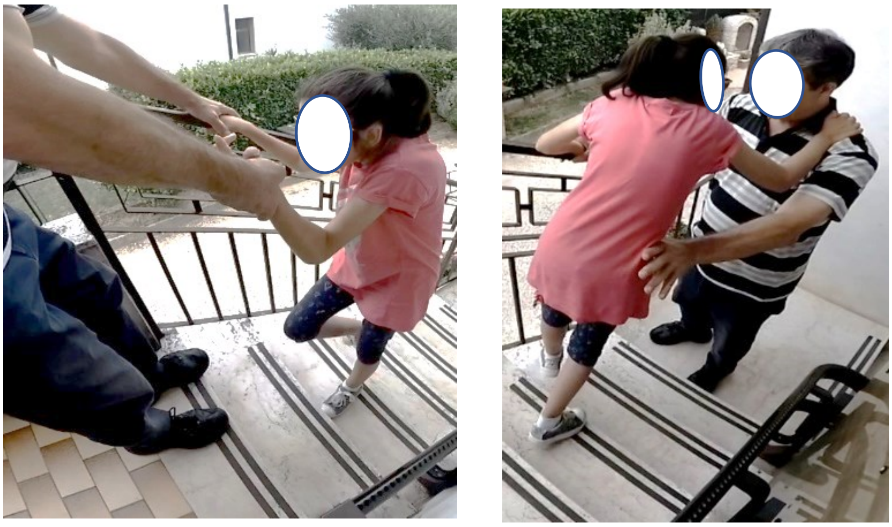

Figure 6a,b

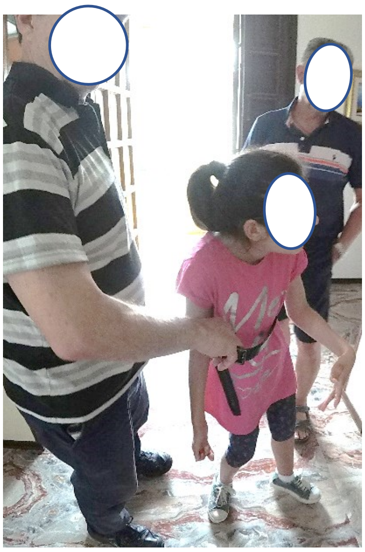

2. During your daily walks together, let her walk alone on uneven terrain (grass, dirt roads, slight ups or downs, etc.) using a safety belt placed under her armpits and kept from the front. The belt should only be used in case of need if she loses her balance (see Figure 7). Take a walk together for at least 20 minutes every day.

Figure 7

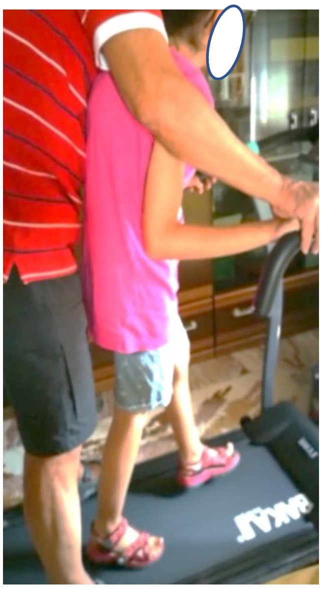

3. Have her walk on the treadmill every day, trying to achieve 20 continuous minutes of exercise. To help her, stand behind her by placing your feet on the side edges of the treadmill. In this way, you can help her with your hands to hold on to the appropriate supports (see Figure 8).

Figure 8

4. Have her lie down on a bed or carpet with a sheet placed horizontally under the thoracic part of her back. With your hands, slowly, bring her elbows as far as possible from the trunk and bring the backs of the hands closer to the bed, as shown in the evaluation. To reach the maximum stretch, it is necessary to bring the arm to the limit of these movements (when you feel that they are resisting) and, at that point, wait a few seconds for the muscles to relax, allowing you to gain a few more degrees of movement. Once you have reached the maximum position, put the part of the sheet that comes out from under her trunk around her elbow and her hand and, without allowing the arm to return towards the trunk, tuck the remaining part of the sheet into the bed or under the mattress so that it keeps her arm in the position you made her reach (see Figure 9). Do the same for both arms. Initially, she will struggle to tolerate this position, so have her hold it for a few minutes and gradually increase the duration of the sessions until she remains at least 20 consecutive minutes.

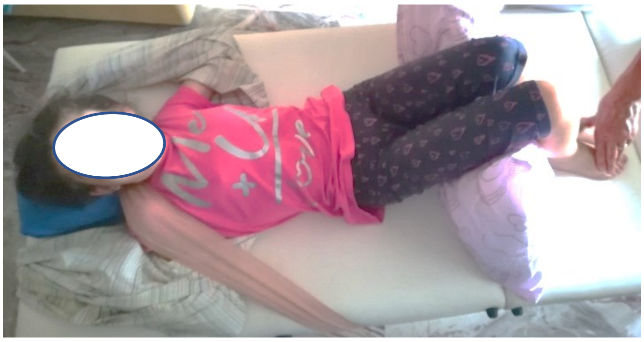

Figure 9

5. Have her lie prone on the carpet or bed with her arms leaning on a roll made of towels of 20 - 25 cm in diameter and placed obliquely so that the part of the roll at her left is further forward than the one on her right (see Figure 10). Ensure that she is leaning on the roll only with the arms and not with the trunk to encourage the spine extension. When she is resting in this position, bring her right foot behind her left knee. You can add a pillow or bandage to keep the right leg in place. Have her maintain this position as much as possible during moments of rest. Try to reach at least 20 minutes per session and do at least one per day.

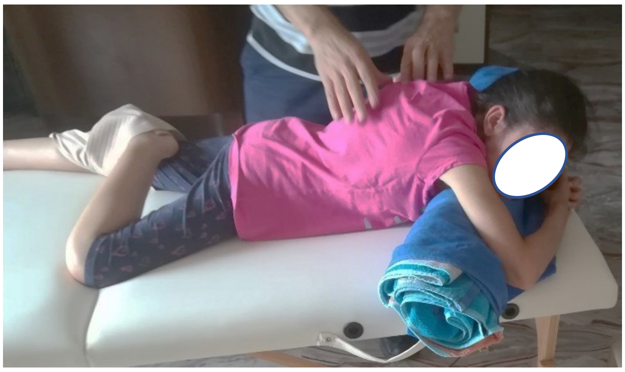

Figure 10

Participant no. 3

1. Have her sit on the sofa next to the armrest with her left elbow leaning on the armrest so that she can watch television. Ensure that the armpit and chest are lifted from the sofa to lengthen the left side of the trunk. This position is perfect for her, and she should keep it for as long as possible, preferring it to her favored sitting posture with crossed legs on the sofa. Next, add a rolled towel between the arm and the head so that the head, leaning on it, remains flexed to the right (see Figure 11). Try to have her hold this position for at least two 20-minute sessions a day. This posture can also be used in the center for moments of rest.

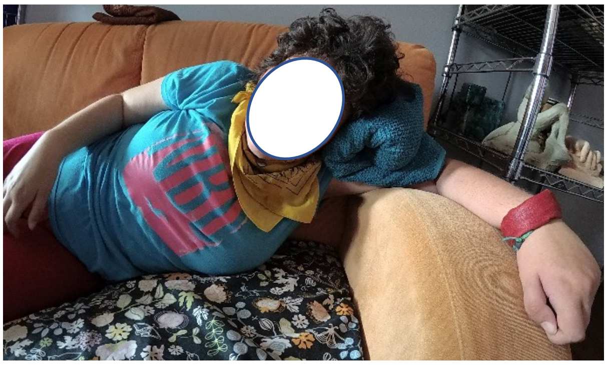

Figure 11

2. Have her stand leaning against her chair with her left hand attached to a cane and her right hand attached to the chair handle (see Figure 12). Let her control her balance and keep herself on her feet. Help her (only if necessary) to keep her hands in place. Have her hold this position for 5 sessions a day of 3-5 minutes each. This posture exercises the maintenance of an upright position and the use of the hands as well as training the muscles of the trunk asymmetrically, counteracting scoliosis.

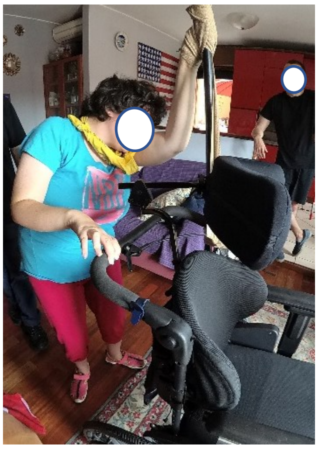

Figure 12

3. Make her walk giving her as little support as possible and only holding her in the right hand (see Figure 13). Let her stand up and prevent her from leaning on your hand. Give her time to walk all the way independently, always walking towards something motivating. For example, have her walk to the kitchen table every day to go and eat and, more generally, whenever you have the opportunity (short journeys like this, at least 6 times a day). Never forget to compliment her and tell her how happy you are to see her walk alone and stand up. This activity can also be carried out in the center.

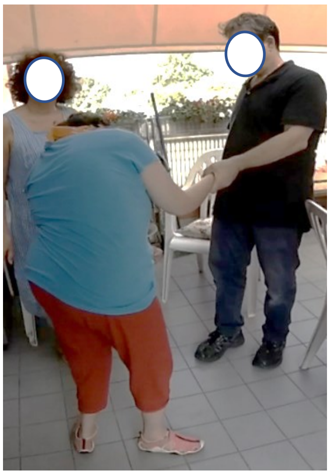

Figure 13

4. Whenever you approach her (e.g., to talk to her, show her something, feed her, etc.), do it from her right so that she is motivated to actively turn her head to the right. For example, have her watching television sitting on the sofa in the living room so she will turn her head to the right to watch.

Participant no. 4

1. Have her lie on the sofa on her right side with her right elbow leaning on the armrest. You can use a towel or small blanket wrapped around the elbow and tucked into the sofa to hold the elbow in place. Make sure that her back and pelvis are attached to the backrest. She can maintain this position even for prolonged periods (up to 30 consecutive minutes) by stretching the muscles of the right side of the trunk, counteracting the rigidity of the spine. When she has calmed down and is relaxing, you can increase the elongation by gently moving the pelvis towards the other armrest.

2. Have her lie on her back on the sofa with her hips and knees semi-flexed and rotated to her left. Use a pillow between the legs to keep them slightly apart. Ensure her shoulders and pelvis are approximately parallel and her right shoulder is leaning on the mattress. You can add a towel tucked into the sofa cushions to hold the right shoulder in place. Add a pillow on the legs behind the knees to maintain rotation. Have her hold this position twice a day for at least 10 minutes each

to lengthen the anterior oblique muscle chain of the trunk by counteracting the rotation of the spine induced by scoliosis.

3. Have her lie on the coffee table in the living room on a blanket with her legs bent out from the short side and held apart by a pillow to stretch the flexor muscles of the hips that risk shortening. In this position, offer her a cartoon to look at her left and help her rotate her head to see it. While she is turned to look at her cartoon, use one hand to tilt her head to the left while, with the other hand, gently push her shoulder towards her pelvis, away from her ear. Gradually and giving her time to relax, try to move the head away from her shoulder as much as possible by stretching the muscles on the right side of her neck. Try to do two stretches a day like this for 10 minutes each.

4. Have her sit on the edge of the table with a folded towel under her left buttock and her right leg tucked under her left thigh. Help her keep herself straight by giving her support primarily to her left hand and encouraging her to straighten up by giving her lots of compliments when she does it. Have her hold this position every day while interacting with her. Initially, you can do two or three sessions a day of a few minutes each. Then, progressively try to lengthen the time spent sitting independently.

5. Have her stand leaning against the stairs handrail with her hands while looking at a cartoon. Try to let her hold this position on her own by helping her stay attached to the handrail and vocally remind her to stand alone. Have her have two 5-minute sessions a day standing in this way. Initially, she will hold this position for only a few seconds. When she comes off or gets tired, let her rest for a while in your arms (30 seconds), then tell her to stand a little longer and help her to resume the position. Give her many compliments when she is holding on by herself.

Participant no. 5

1. Choose a pair of her shoes to add a 1cm elevation under the entire sole of the left shoe. She will mostly have to wear these shoes for the six-month program, so we recommend that you choose a comfortable pair that comes above the ankle to offer good support. Have her wearing these shoes for most of the day.

2. Take the daily walk with her without giving her any support but using the safety belt instead (see Figure 14). While walking, the belt should not offer her any support, except in the case of a fall. Let her manage her body alone and decide where she wants to go by simply following her and eventually indicating a goal. Walk like this with her for at least 20 minutes a day.

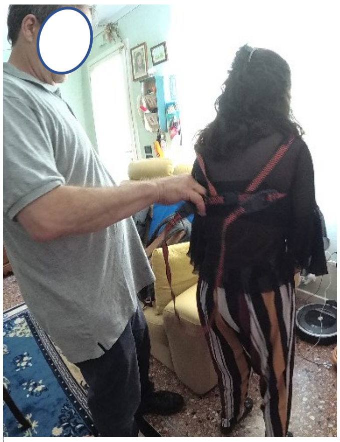

Figure 14

Dance with her to rhythmic music she likes while she is wearing her seat belt. Pull her hand from behind to the left side of the seat belt and move with her in rhythm by unbalancing her in all directions and letting her adjust her balance by herself (see Figure 15). You can also dance with her in front of her mirror by standing behind her. You can gradually increase the speed and amplitude of the movements you make during the dance. Dance together for at least 15 minutes a day.

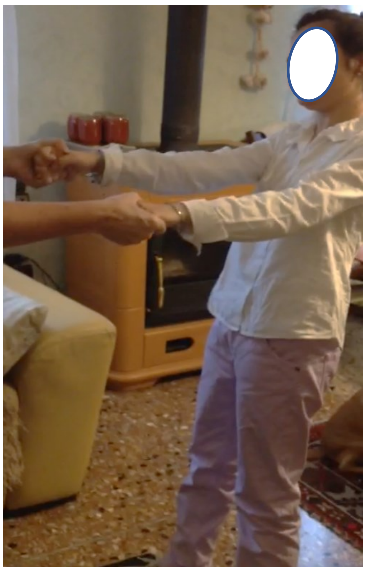

Figure 15

3. Have her sit in a chair without a back and place an L-folded towel under her buttocks and left thigh so they are elevated. In this way, she will use the muscles of the left side of the trunk more to stay upright, counteracting scoliosis (see Figure 16). Make her sit like this whenever you have the opportunity (while she looks at the cartoons, while you comb her hair, etc.) and in general at least twice a day for 10 minutes each. This exercise can also be done in school.

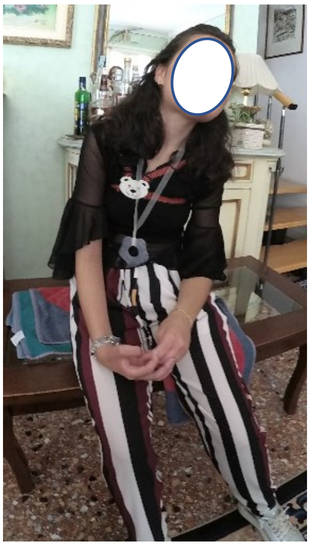

Figure 16

4. When she needs to rest, she can lie down with her arms resting on the half roll she has at home to stretch the front muscles of the trunk and shoulders (see Figure 17). Ensure she only leans her arms and head on the roll while the trunk must remain slightly raised from the surface to allow elongation. In this position, she can receive a relaxing back massage to facilitate muscle relaxation.

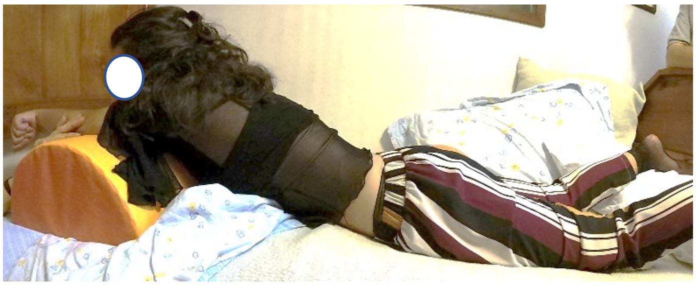

Figure 17

5. At home, she can sit in the Swedish chair (the one with the weight on the knees) while she looks at the cartoons with her hands on the table just behind her back so that she has an incentive to extend her trunk to lean on it (see Figure 18). This position can be maintained with little supervision. Sit her like this for at least 15 minutes a day.

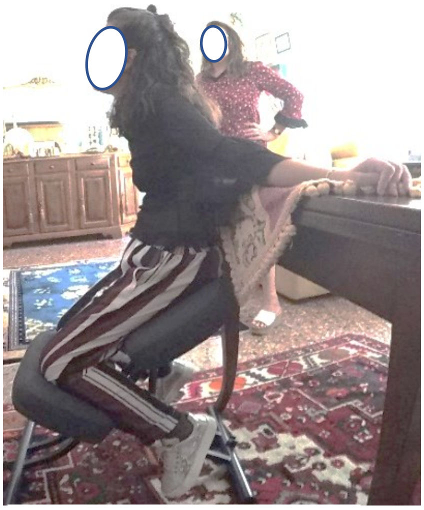

Figure 18

Participant no. 6

1. Each time she sits on a chair different from the postural one you have, have her sit on an L-folded beach towel to raise her buttocks and right thigh, helping her maintain a more upright trunk posture. Then, for optimal correction, bend her left leg under her right thigh (see Figure 19).

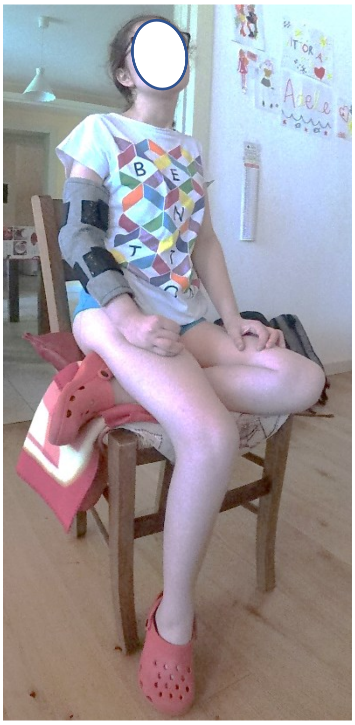

Figure 19

2. Have her stay on her knees with her hips extended to strengthen the muscles that extend the trunk and improve balance. Initially, let her hold the position for short durations (a few minutes) and gradually increase the time she spends on her knees to perform sessions of 10 minutes each. Try to get her to do at least one session a day. Play videos she likes on television and have her kneel on her carpet behind the sofa so that she can only see the screen if she holds the position (see Figure 20). While maintaining this posture, she should wear her elbow braces.

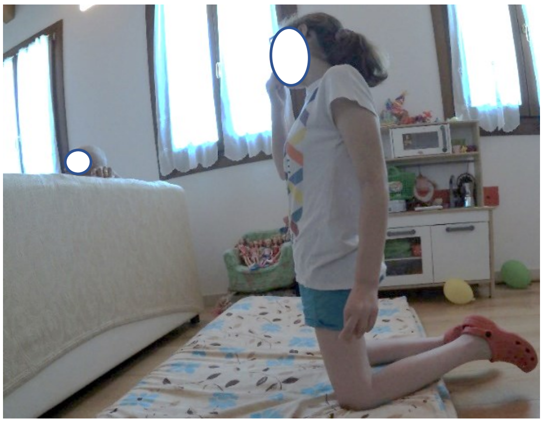

Figure 20

3. Let her walk around the house without the left slipper every time you have the opportunity and, in general, at least 20 minutes a day. In this way, with each step, she will activate the muscles of the right side of the trunk, working against scoliosis (see Figure 21).

4. Have her walk for at least 15 minutes a day independently on uneven ground (e.g., tall grass, gravel, dirt roads, etc.) (see Figure 22).

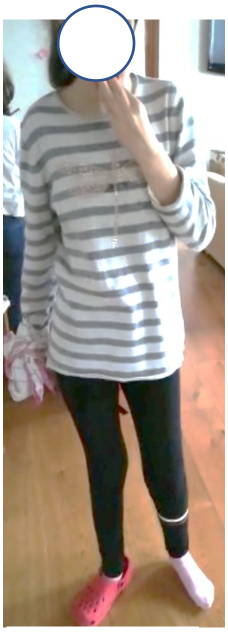

Figure 21

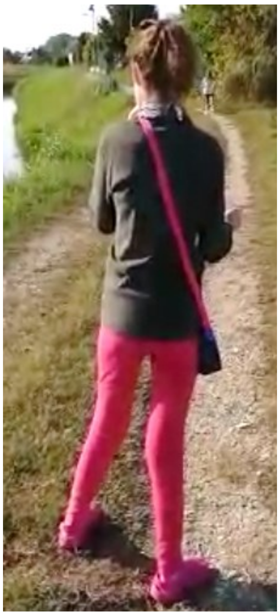

Figure 22

5. Have her take the stairs up and down at least twice a day. Always offer her one-sided support from the front. To go up, tell her to hold onto the handrail with one hand while you hold her other wrist. Let her make the effort, do not pull her. To go down, have her lean with one hand on the handrail and the other on your shoulder. Use your hands to help her hold her hand in position (see Figure 23a,b).

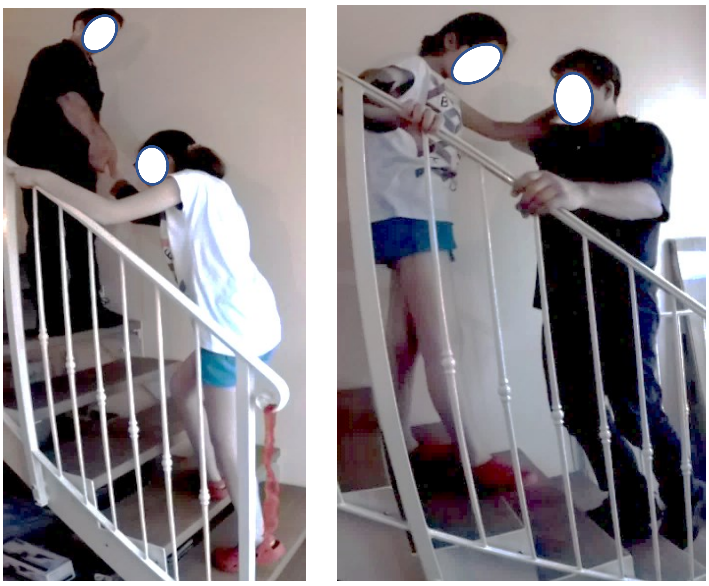

Figure 23a,b

Participant no. 7:

1. Have her walk for at least 60 minutes a day (even non-continuous, e.g., 3 walking sessions of 20 minutes each), including walking outdoors with her parents and at home on the treadmill (which, after some training, can be done by her independently with only little supervision). When walking outdoors, try to prefer more difficult or uneven terrain (e.g., slopes, grass, gravel, stones, etc.). To get her used to walking on the treadmill, start the program with a few minutes of walking and gradually increase the exercise duration until you have sessions of at least 20 minutes. Always make her walk while watching her favorite cartoons to keep her motivation high (you can choose a cartoon that she can watch ONLY when she is walking). Initially, it will be necessary to get her used to the fact that the cartoons are played only if she is walking. Therefore, if she stops, it will be required to stop the reproduction and explain to her that, to start them again, she will have to start walking again. When you have reached the complete duration of the activity, begin to gradually increase the treadmill's incline to increase further the amount of activity performed.

2. Have her sit on an inflatable wedge pillow oriented diagonally forward and left (the right buttock must be higher than the left). You can use any type of chair in this way, but the best result is obtained using a stool (without backrest) (see Figure 24).

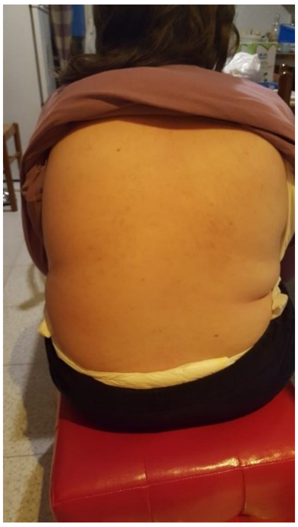

Figure 24

3. Have her walking upstairs and downstairs at least five times a day (e.g., asking her to bring something located upstairs) (see Figure 25).

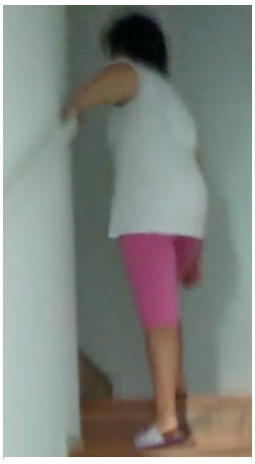

Figure 25

4. Try to ride a bike with her for at least 15 minutes and always with motivation (e.g., visit grandparents, get ice cream, etc.) (see Figure 26).

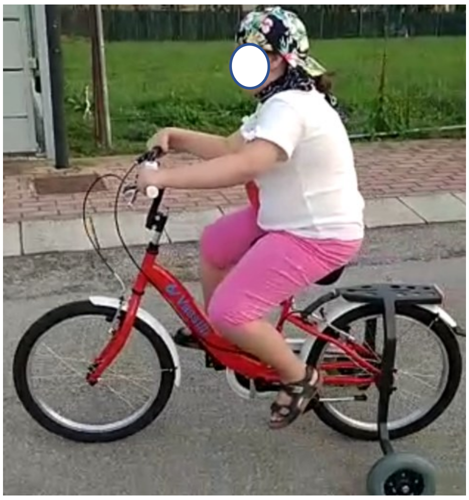

Figure 26

5. Let her help you set the table for dinner by asking her to take all the needed objects (e.g., plates, cutlery, glasses, etc.), placed above her head (as high as possible), one at a time. Try to get her to take at least 20 items at each meal by adjusting the quantity basing on how long she takes to carry out the entire routine of setting the table. At school, she can practice in the same way by having a shelf for her school materials (e.g., pencil case, diary, notebook, etc.) and asking her every morning to prepare her desk and, at the end of the lesson, to put things back on the shelf. Another way to practice this skill at school is to attach (above her head) several pictures that she needs for her activities to the wall whit velcro so that she has to go and get them to start the chosen activity (see Figure 27).

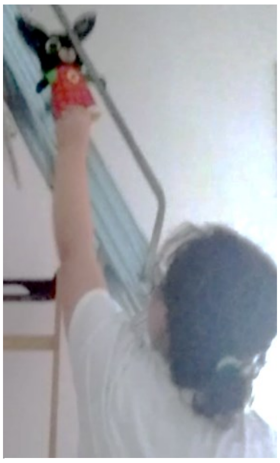

Figure 27

Participant no. 8:

1. Insert an L-folded towel inside the chair she uses to sit at the table to elevate her buttocks and left thigh. This rise should be at least 2cm (becoming 4cm below the left buttock where the wipe forms the corner). Have her sit like this every time she uses this seat. Make her sit in the same way in her postural chair by putting the same L-folded towel inside and slightly raising the chair's rear legs so that the seat is tilted forward (see Figures 28a,b,c).

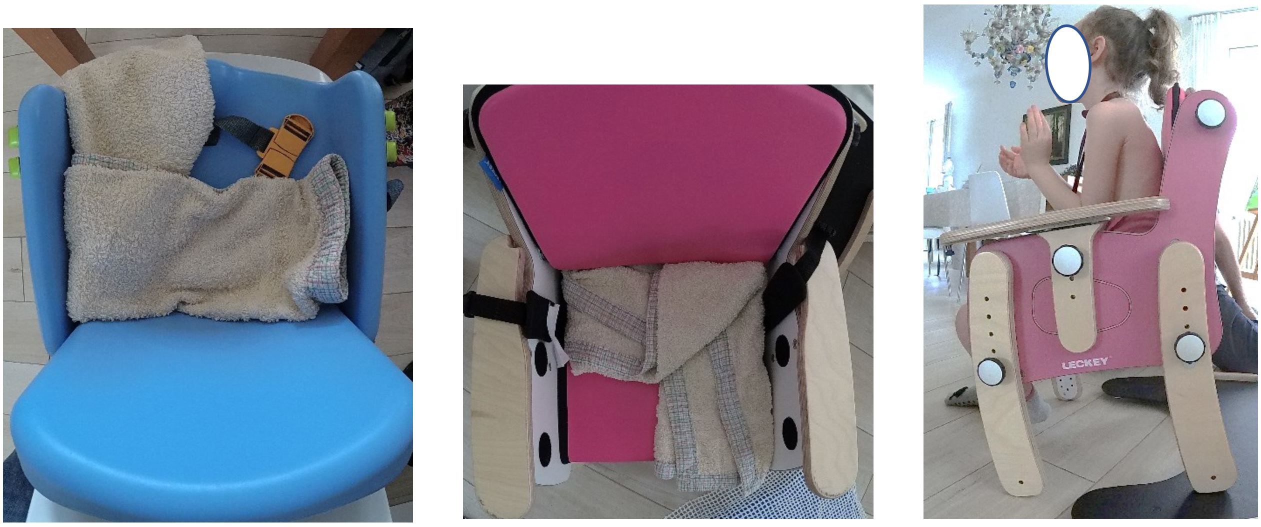

Figure 28a,b,c

2. Add an insole inside her left shoe under the heel every time she walks with the shoes. The elevation you add should be between 0.5cm and 1cm. At home, she can also walk wearing only the left shoe and a non-slip sock on the right.

3. Have her sit on the edge of the table on a wedge-shaped cushion oriented forward and to the right (left buttock higher than right) and with her left knee rotated to the right and bent to place her left foot on the table beside her. Sit her like this for at least 15 minutes a day. Initially, start with a few minutes and gradually increase the duration of the exercise. Do something nice with her while she holds this position to motivate her to stand straight with her back. If she needs it to improve her stability, you can add a sandbag over her left knee to stabilize it against the tabletop (see Figure 29).

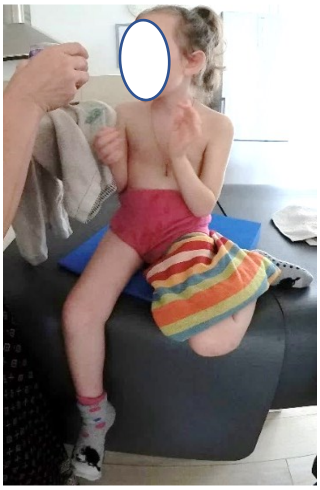

Figure 29

4. Create obstacle courses in the house that she will face to cross the room and reach something pleasant (dad, mom, brother, dog, cuddles, videos, snacks, etc.). You can use heavy objects she will have to go around or some objects to leave on the ground, which she will have to climb over. If you have a carpet, you can hide under it many small objects of different shapes and textures to create an uneven floor to walk on. For safety, use an underarm belt to give her support in case of falling. This belt is to be used only in case of need and safety. Go through one course a day with her for at least 15 minutes.

5. Have her sit inside the balance plate on top of a round cushion and a wedge to sit slightly tilted forward and to the right (elevation of the left buttock). In this position, place her left hand in the trousers so that she has to control the movements of the plate with the muscles of her abdomen. Have her sit like this every day for at least 15 minutes per session. Initially, you can reduce the exercise duration and gradually increase it (see Figure 30).

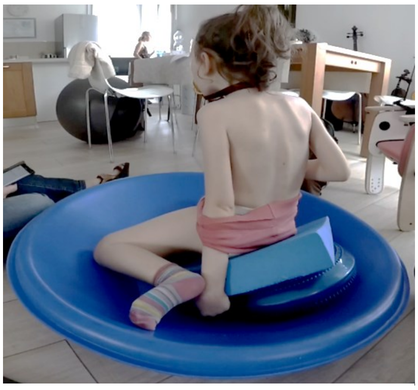

Figure 30

6. Whenever she has to get up or sit down, let her do it as independently as possible, always giving her a reason to do it (e.g., “Come on, let’s go and call Dad for dinner”). The procedures below should be done by giving her as little support as possible, letting her actively carry out the movements.

   a. Stand up: kneel in front of her placing your knees on the tips of her feet to stabilize her feet on the ground. Ask her to stand up and explain why she has to do it. Then take her right hand with your left and exert a slight pull (not enough to “pull her up”) forward (not up). Let her lean forward slightly and hold her wrist firmly. At the same time, if necessary, you can stabilize her left knee by holding it still with your hand (the arrows in Figure 31 represent the direction in which you need to pull her arm slightly and push on the upper part of the knee to help her get up). Give her time to organize the movement and compliment her when she gets up. Again, the traction forwards and not upwards is fundamental.

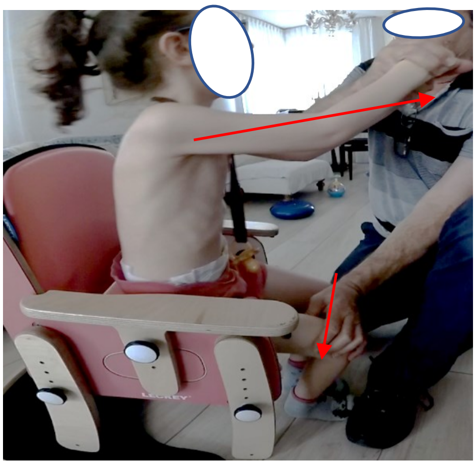

Figure 31

   b. Sit down: have her sit with a chair behind her so that she can feel her against her legs. Help her keep her balance by taking one or both hands, ask her to sit down, and give her time to organize the movement. To facilitate sitting, press lightly with your free hand against her pelvis, pointing the pressure in the direction of the angle formed by the seat and the back of the chair (red arrow – Figure 32).

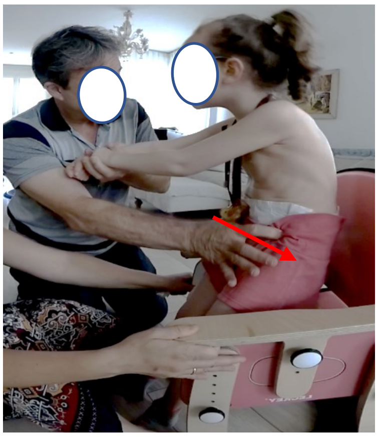

Figure 32

Participant no. 9

1. Add a two-centimeters elevation under the left shoe every time she walks to correct the leg dysmetria and enhance the muscle activation on the right side of the back through balance reactions during walking and standing activities.

2. Have her sit asymmetrically on a stool on an L-folded towel that elevates her buttocks and left thigh and bending the right leg under the left thigh (see Figures 33a,b). Have her keeping this position while watching TV for at least 20 minutes a day. Meanwhile, to exercise her hand function, the phone/tablet was held in front of her, over her shoulder, so she tried to reach it with her right hand. She will go out of balance at every attempt of reaching and use the trunk muscles to regain balance.

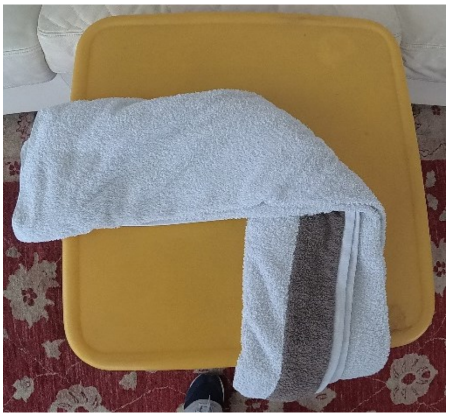

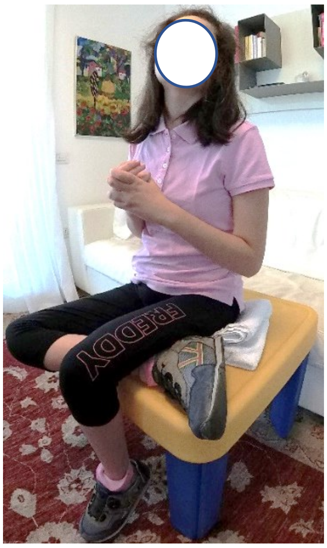

Figure 33a,b

3. When she has a seizure, she fell asleep for few hours. In those moments, let her rest inside a “U” shaped pillow on her left side to stretch the muscles of the right side of her trunk. Place the pillow under the tip of her scoliosis curve (see Figure 34). The cushion’s thickness should be as high as possible to allow maximum stretch but remaining comfortable enough to let her sleep.

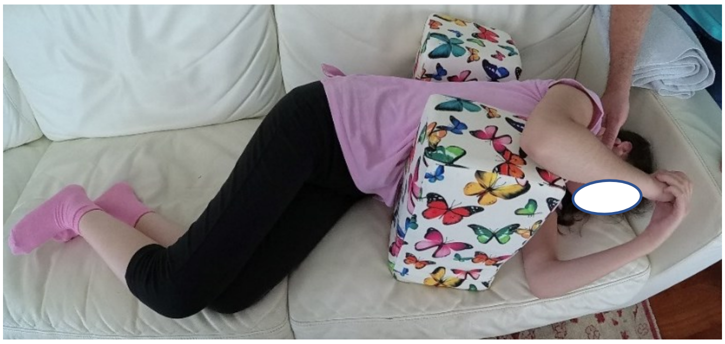

Figure 34

4. Have her walk in the park with a safety belt around her trunk (see Figure 35) every day for at least 45 minutes. During the walk, let her could move freely and decide where to go. Walk with her without providing any support, except in case of an epileptic seizure or falling risk of falling. As the park presents some small hills, walk with her along the side of the hill, counterclockwise, so that her left foot is uphill (higher) and her right foot downhill (lower), activating her trunk's left side muscles at each step. Try to minimize the time she spent sitting on the benches at the park, and if she needed to sit, use the position suggested in activity 2.

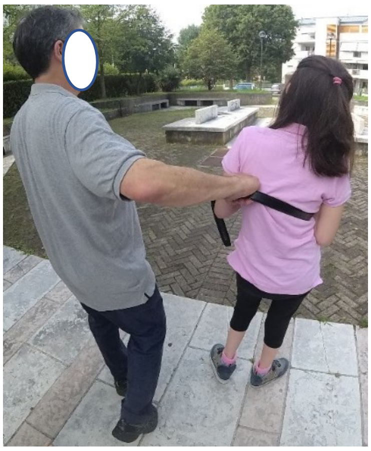

Figure 35

5. Walk up and downstairs with her, providing help to hold onto the railing with one hand and maintaining her other hand. Avoid pulling her to make her actively perform the steps (see Figure 36). Try to walk up and downstairs with her every time you go out of your house and, in general, at least twice a day for at least one flight of stairs.

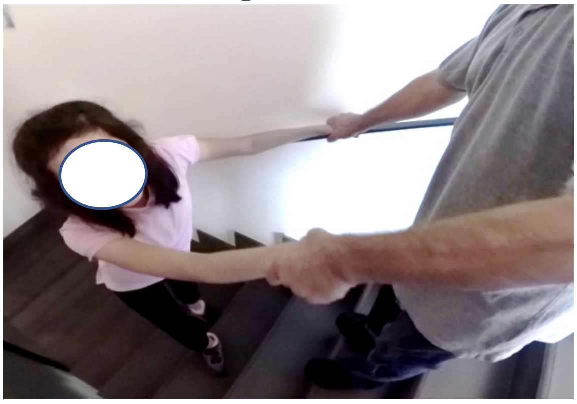

Figure 36

Participant no. 10

1. Have her sit on the left front corner of the chair with her left knee resting on the rectangular sofa cushion attached to the left legs of the chair. Ensure that the left hip is perpendicular to the ground or slightly extended (with the knee resting further back than the pelvis). To provide greater stability, you can secure the right hip to the right rear leg of the chair and the left leg to the sofa cushion. If needed, help her keep her back against the chair's backrest (see Figure 37a,b). This exercise activates the muscles of the right side of the trunk, counteracting the progression of scoliosis. Have her hold this position every day initially for 2 sessions of a few minutes (5-10) and then gradually increase the exercise duration until you have sessions of at least 20 minutes each.

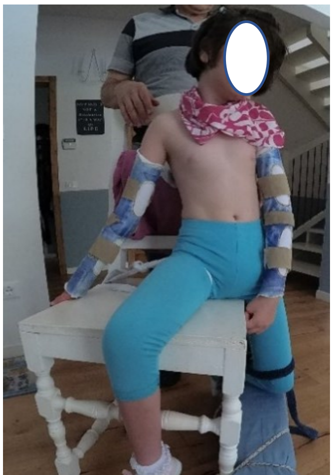

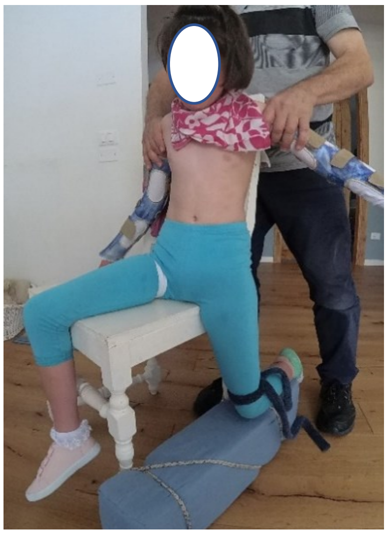

Figure 37a,b

2. Exercise 1 is very demanding for her and, after doing it, she can rest lying in a prone position on a carpet with her arms leaning on the rectangular cushion of the sofa or on a roll made of towels of 20 - 25 cm in diameter placed obliquely so that the part to her left is further forward than the one to her right. Ensure that only her arms (not the trunk) are on the pillow to encourage the spine extension. While she is resting in this position, bring her right foot behind her left knee (see Figure 38). You can add a pillow or bandage to keep the right leg in place. Have her hold this position as much as possible at times when she is resting. Try to reach at least 20 minutes per session and do it at least once a day, but the longer she rests in this position, the better.

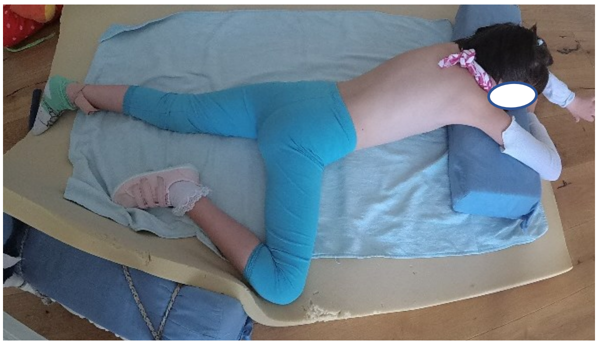

Figure 38

3. Whenever she has to get up or sit down, let her do it as independently as possible, always giving her a reason to do it (e.g., “Come on, let’s go and call your brother for dinner”). The procedures below should be done by giving her as little support as possible, letting her actively carry out the movements.

   a. Stand up: kneel in front of her placing your knees on the tips of her feet to stabilize her feet on the ground. Ask her to stand up and explain why she has to do it. Then take her right hand with your left and exert a slight pull (not enough to “pull her up”) forward (not up). Let her lean forward slightly and hold her wrist firmly. At the same time, if necessary, you can stabilize her left knee by holding it still with your hand (the arrows in Figure 39 represent the direction in which you need to pull her arm slightly and push on the upper part of the knee to help her get up). Give her time to organize the movement and compliment her when she gets up. Again, the traction forwards and not upwards is fundamental.

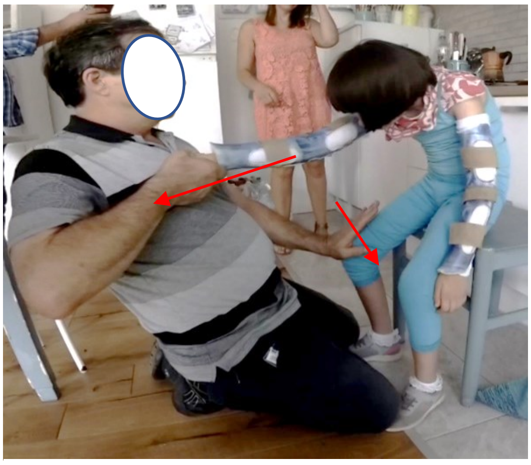

Figure 39

   b. Sit down: have her sit with a chair behind her so that she can feel her against her legs. Help her keep her balance by taking one or both hands, ask her to sit down, and give her time to organize the movement. To facilitate sitting, press lightly with your free hand against her pelvis, pointing the pressure in the direction of the angle formed by the seat and the back of the chair (red arrow – Figure 40).

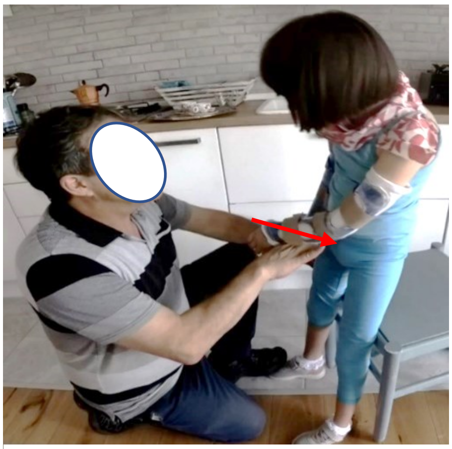

Figure 40

Participant no. 11

1. Have her sit without support to her back or feet on the inflatable wedge pillow oriented diagonally forward and to the right (so that her left buttock is higher than the right) (see Figure 41). Try to keep her in this position for at least 20 minutes a day. To reach the complete duration of the exercise, start with short sessions at different times of the day (e.g., 4 sessions of 5 minutes) and progressively lengthen the duration of the sessions.

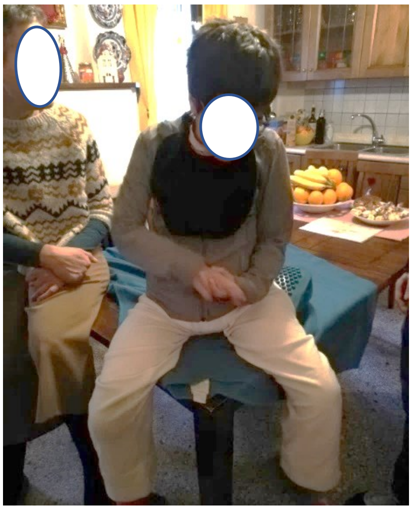

Figure 41

2. Walk with her on the treadmill every day at 1km / h and without incline, supporting her from behind, under her armpits. Place your feet sideways to the tape (see Figure 42). Start with three one-minute sessions alternating with a one-minute break (1min of exercise, 1 minute of rest, x3). Those who walk with her should wear a lumbar band to protect their muscles. In addition, you can add front supports to the treadmill to allow those who walk with her to hold on.

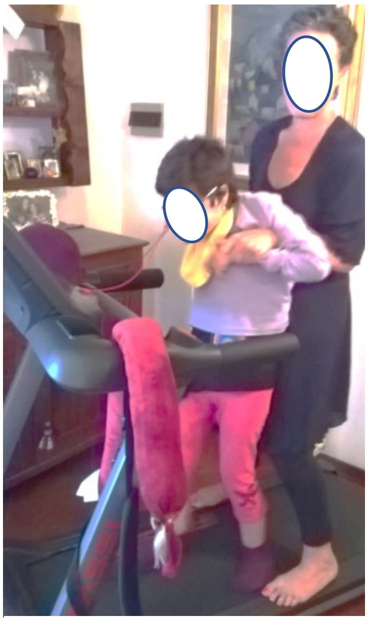

Figure 42

3. Her day is quite busy for her, and for this reason, she spends a lot of time lying down or sitting at home. Below are several positions in which she can rest after the activities proposed above and during the day to maintain correct postural hygiene. You can have her hold these positions in rotation throughout the day as she relaxes. Each of the following positions must be maintained for at least 20 consecutive minutes to allow muscle stretching. There are no side effects so that these positions can be maintained as long as possible and used in her daily routine.

a. She can rest lying prone on the sofa with her arms on the sofa's armrest. Ensure she is leaning only her arms and elbows on the armrest and not the trunk to encourage the spine extension. While she is lying down in this way, slightly bend her left knee and insert her left foot into a cuff made off her right pant leg at knee level (see Figure 43) so that it stays in position, encouraging the trunk right-side extension.

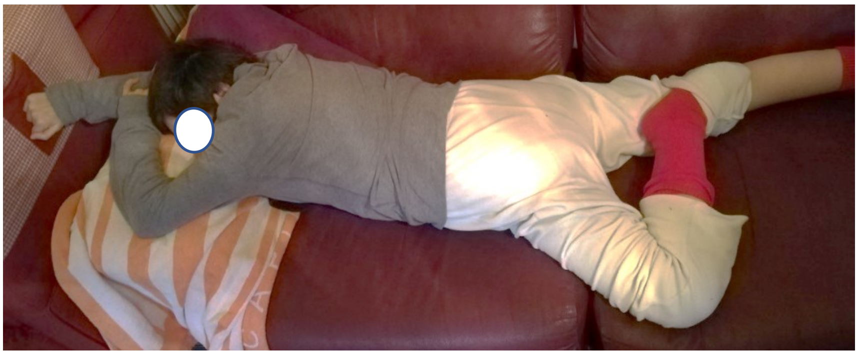

Figure 43

b. While she is sitting in her chair, add a square and stiff pillow and a roll pillow under her right arm and position her legs so that both knees point to her right to encourage the extension of the right side of the trunk. Ensure her pelvis is as close as possible to the chair's left armrest (see Figure 44).

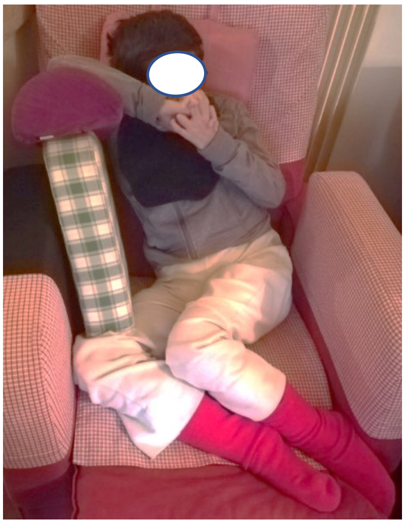

Figure 44

c. To prevent scoliosis progression and stretch the muscles of the right side of the trunk, she should rest lying on her left side inside a "U" shaped pillow. The pillow should be placed under the apex of her scoliotic curve. This position should be maintained without a pillow under the head. Initially, to get her used to, you can use a low pillow and gradually reduce its thickness until it is completely removed. Next, add a cushion between the legs to keep them in the correct position (see Figure 45).

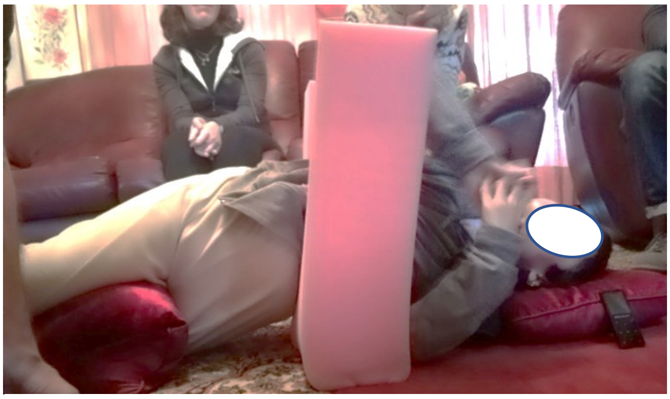

Figure 45

Participant no. 12

1. Have her stand in front of the sofa (for safety) while holding the handlebars of her posture system with her hands and looking at something interesting in front of her (see Figure 46). Have her hold this position every day 2 times a day, trying to reach 10 consecutive minutes. Start with short sessions at different times of the day (e.g., 4 sessions of 5 minutes each) and progressively lengthen the duration of the sessions and reduce their number (e.g., 3 sessions of 7 minutes each).

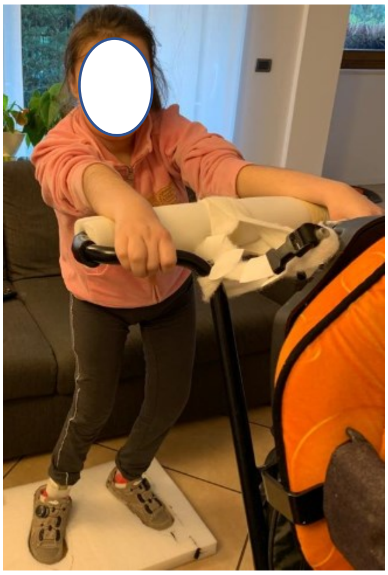

Figure 46

2. Have her sit on the outer edge of the sofa armrest on an air cushion with her left hip extended and her knee resting on a stool or pillow. Ensure her left hip is fully extended (at least 180 °, even better if a little more) (see Figure 47). If necessary, you can support her right wrist to help her hold the position. To strengthen the right side back muscles, she should sit like this as much as possible. Try to have her hold this position for at least 20 minutes a day. To reach the entire exercise duration, start by doing several sessions of a few minutes and gradually increase their duration.

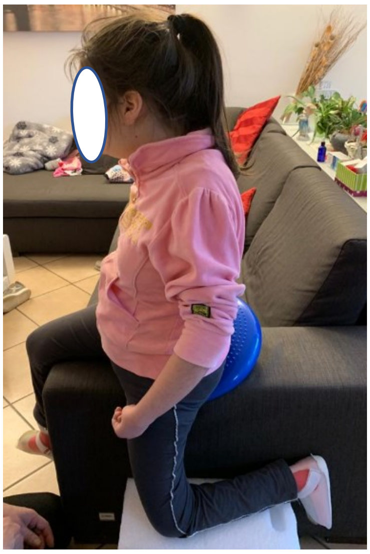

Figure 47

3. When she needs to rest, is tired, or after activities, she can do it lying prone on the sofa with her arms on the armrest. When maintaining this position, make sure she is leaning on the armrest with only her arms and head while her chest should remain slightly off the mattress. In this way, she will stretch the anterior muscle chain of the trunk, counteracting the kyphosis. While she is lying down in this position, bend her right knee and hip to encourage the stretching of the muscles on the left side of the trunk (see Figure 48). This position can be maintained for long periods without side effects. The more she will be able to relax in this position, the more her muscles will stretch. Have her hold this position at least 20 minutes once a day when she is tired or wants to rest.

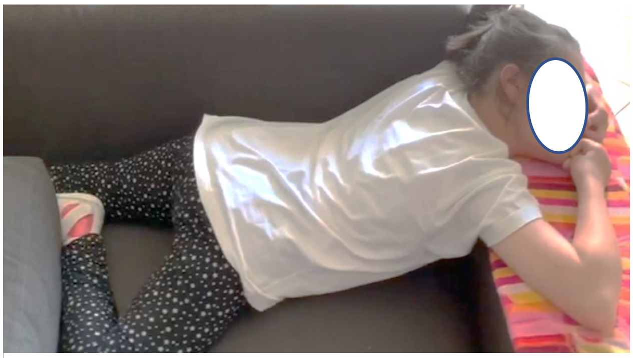

Figure 48

4. To maintain the spine and shoulders mobility, she can rest on her back with her hands above her head and her arms kept away from her body by two towels, as shown in the evaluation. To reach this position, take 2 large towels and join them by rolling them together to create a long roll. Then have her lie on the sofa and place the roll under the apex of the kyphosis. Slowly bring her elbows as far away from the trunk as possible and bring the backs of her hands and elbows closer to the surface of the sofa. To reach the maximum stretch, it is necessary to bring the arm to the limit of their movements (when you feel that they are resisting) and, at that point, wait a few seconds for the muscles to relax, allowing you to gain a few more degrees of movement. Once she has reached the maximum position, put the part of the towel that comes out from under her torso around her elbow and hand and, without allowing her arm to return towards the torso, tuck the remaining part of the towel under the sofa cushions so that it keeps her arm in the position (see Figure 49). Do the same thing for both arms. If this position is difficult for her to tolerate at the beginning, have her hold it for a few minutes and gradually increase the duration of the sessions until they last for at least 20 consecutive minutes. If she tolerates it well, you can make her stay even for longer durations. There are no contraindications. The longer she holds this position, the more her shoulder muscles will stretch, preventing further muscle shortening and counteracting kyphosis.

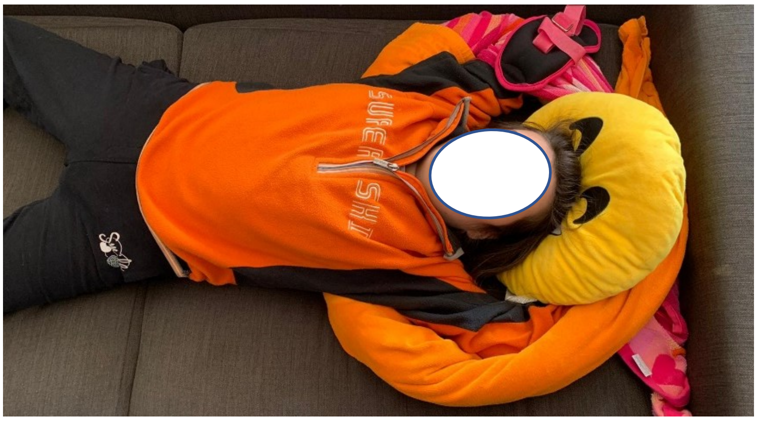

Figure 49

5. Have her stand with her back against the wall. Then, to prevent her feet from slipping, place them inside the specially created styrofoam shape (see Figure 50). Have her stand like this for at least 20 minutes. Start with short sessions and progressively increase their duration.

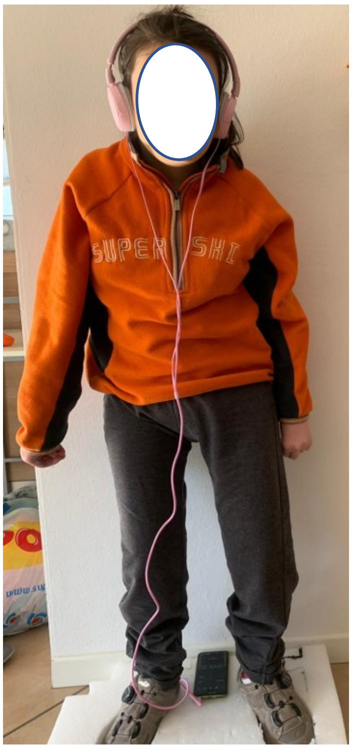

Figure 50

Participant no. 13

1. When she sits on the sofa, she tends to assume an incorrect posture that favors scoliosis and kyphosis progression. Therefore, you should avoid sitting on the couch leaning back. To relax while sitting, we suggest getting her a rigid chair with armrests to which you can add a pelvic belt that holds her pelvis against the corner of the seat (see Figure 51). This belt should be placed at 45° to be effective.

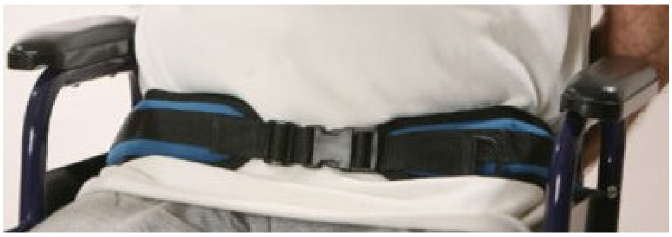

Figure 51

2. Whenever you have the opportunity, have her sit without a backrest on the wedge cushion with the slope oriented diagonally forward and to her left (see Figure 52). The same result can be obtained by making her sit on two beach towels folded in an "L" shape and placed one on top of the other. This should become the position in which she spends most of her time sitting throughout her day. Try to make her keep it at least 30 minutes a day (if they are more, even better!). This position can be maintained unsupervised, but make sure that while she sits like this, her shoulders are well aligned, or her torso is slightly flexed to the left (in the opposite direction of her scoliosis).

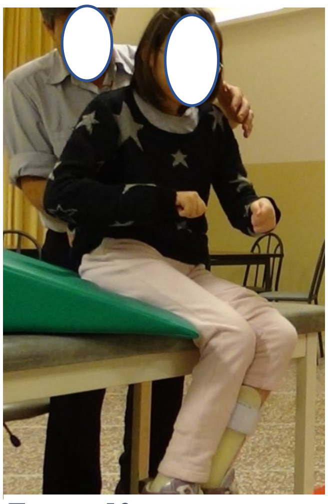

Figure 52

3. Supra-malleolar orthosis (SMO) should be prescribed for her as they offer adequate lateral support to the foot, reduce supination, and allow the maintenance of a better posture in a standing position (see figure 53a,b).

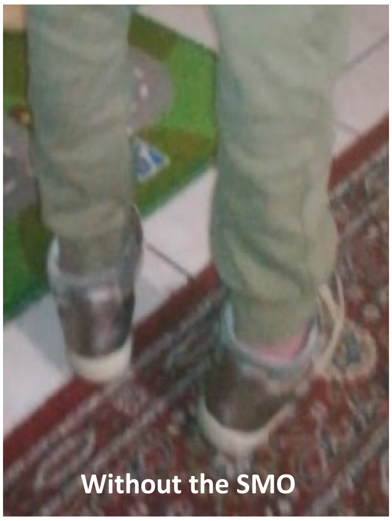

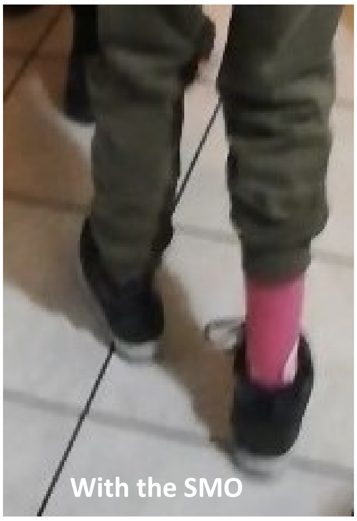

Figure 53a,b

4. When she is tired and needs to relax, she can do so by lying on the sofa on her right side with her right arm and elbow leaning on the armrest. You can add a robe belt that goes around her arm and snaps into the sofa cushions to keep her elbow in place. This position should be held for at least 20 minutes to allow the muscles of the right side of the trunk to relax and stretch. Eventually, to let her be more comfortable, you can add a pillow between the shoulder and the head to lean her head.

5. Create the opportunity to let her walk independently every day for at least 30 minutes (even if not continuously – e.g., 3 sessions of 10 minutes each, if they are more, even better!). Always give her a direction and a goal to walk towards (e.g., go to the car, go home, go for a snack, see a cartoon, find her brother/sister, etc.). Try to make her walk on her own, eventually provide her a little help to start walking by placing only a finger on her shoulder to help her move. She should always walk wearing splints as the abnormal positioning of her foot can cause pain and load asymmetry, which contributes significantly to the development of scoliosis.

6. Try to go up and down at least 2 flights of stairs a day with her as described below. Always motivate her to perform the stairs and wait for her to carry out the movements independently. Always provide as little help as necessary to get her to complete the step by adjusting the support you provide as needed. Try to take advantage of times when she has to go up or downstairs in her daily routine (e.g., when she leaves for school and comes home). Take the time to make her as independent as possible. Gradually it will be easier and easier for her to climb the stairs, and she will reduce the time needed to complete them.

    a. Climb up - Go up the stairs with her by standing in front of her and firmly holding both of her wrists. While holding her wrists, help her shift her weight onto the right or left leg to facilitate her freeing the other leg and making her take the step. To make it easier for her to climb up, after having shifted her weight onto one foot, unbalance her slightly towards you and give her brief and clear indications on what to do (e.g., "Lift your foot!"; "Push on the leg!"; "Climb up!"; Etc.). Compliment her every time she actively participates in a step, and if you have promised her that she will get something at the end of the stairs, remember to give it to her once she has completed the flight.

    b. Go down - Stand in front of her. Hold her firmly with your hand from one wrist and keep her other hand on the handrail (see Figure 54). As for the climb, always give her a reason to go down the stairs and give her clear and precise indications on the movements she must make in each step. To make it easier for her to go down, move her hand forward on the handrail to unbalance her towards you and use your hand holding her wrist to help her manage her balance. Once she is unbalanced, invite her down the step and wait for her to do the movement. It may also be necessary to help her to free one foot by shifting her weight onto one leg. Compliment her every time she gets down a step and always give her as little help as possible to have her going down.

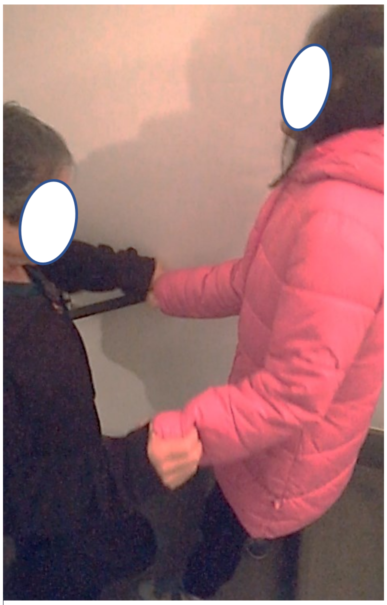

Figure 54

Participant no. 14

1. When she uses her walker at home, add a thickness under her left arm to encourage extension and right flexion of the trunk. Add a compression bandage that presses gently against the apex of the scoliotic curve and rotates her torso counterclockwise against the induced rotation of scoliosis (see Figures 55a,b). The therapist can guide the parents in the correct use of the bandage. Use the bandage and the thickness whenever you know she will spend some time in the walker. However, try to make her walk like this for at least 20 continuous minutes a day.

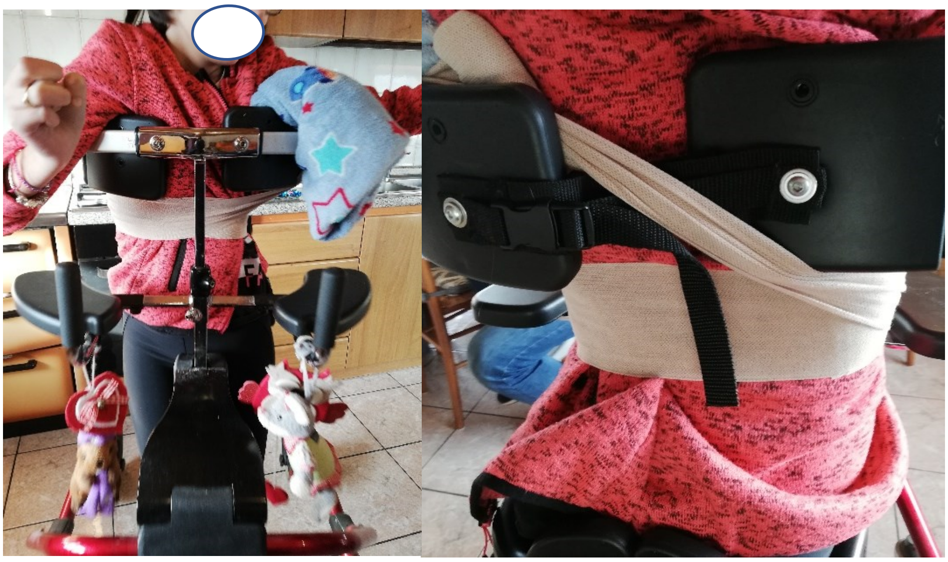

Figure 55a,b

2. To prevent the progression of scoliosis and allow the muscles of the left side of the trunk to stretch, she should rest lying on her right side inside the "U" pillow. The pillow should be placed under the apex of her scoliotic curve. This position should be maintained without a pillow (see Figure 56). Initially, to get her used to the position, she can use a low pillow. Then, gradually reduce the pillow thickness until it is completely removed. To facilitate the relaxation of the muscles, you can add a hot water bottle on the left side of her trunk. She can maintain this position every time she has to rest while lying down. She must hold it for at least 20 consecutive minutes to allow adequate stretching of the muscles. There are no side effects, and if she can, she can even sleep in this position.

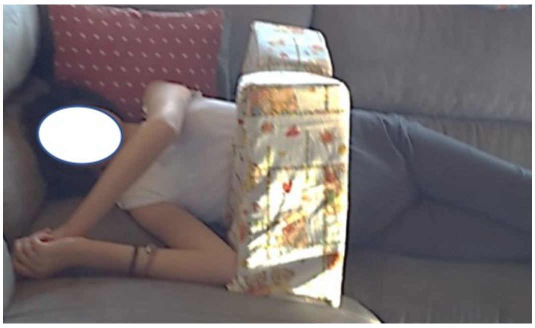

Figure 56

3. Have support added on the outer and rear edges of the left armrest of her chair so that when she leans her elbow on it, it cannot go down, causing her to bend in favor of scoliosis (a bandage was used in Figure 57). When you have added the support, ensure that she always has her elbow on the armrest while sitting in her chair.

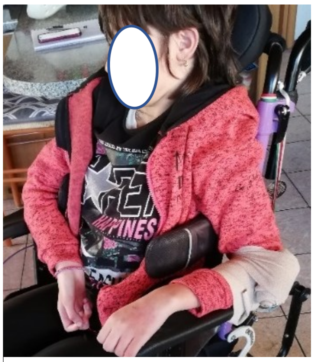

Figure 57

4. Have her sit on the wedge pillow without seatback with her knees oriented slightly to her left and her feet shifted to the right of her knees. Place the highchair to her right and add a rolled-up cushion between the armrest of the highchair and her right side, pushing against the scoliotic curve (see Figure 58). You can help her hold onto the armrest of the highchair with her right hand. She should sit like this for as long as possible, try to make her reach at least 20 minutes a day. To achieve the complete duration of the exercise, start with short sessions at different times of the day (e.g., 4 sessions of 5 minutes) and progressively lengthen the duration of the sessions.

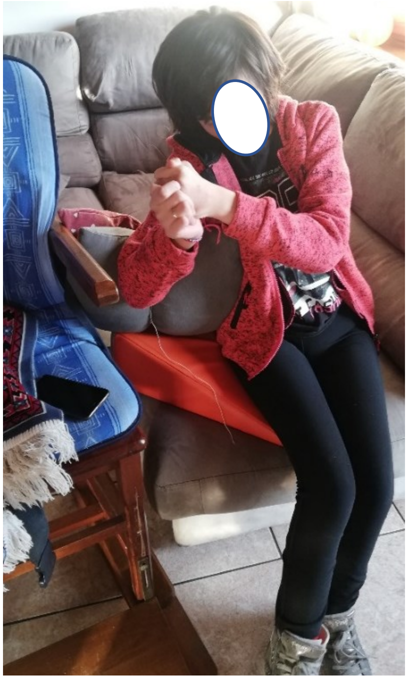

Figure 58

5. To get her to use the muscles on the right side of her torso, sit her with a large towel folded under her right buttock and thigh (so that she is leaning towards her left) and bend her left leg on a small stool placed behind the right leg (see Figure 59). This position should be kept as long as possible until she can maintain her back straight without bending to the left. Try to have her sitting like this for at least 20 minutes a day.

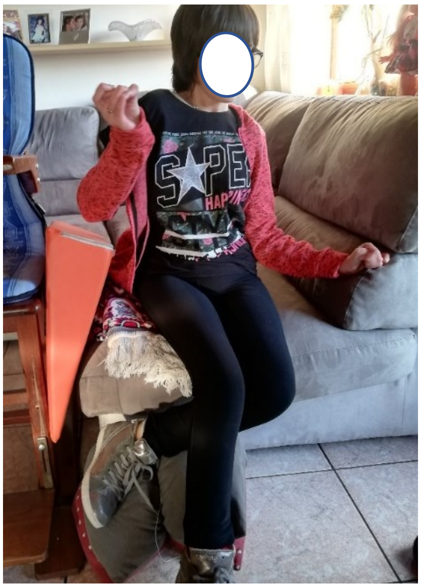

Figure 59

Participant no. 15

1. To strengthen the musculature of the right side of the trunk and counteract scoliosis, have her stay on her knees with the hips extended and a slight rise below the right knee (about 3 cm, e.g., a folded beach towel). While holding this position, her left leg should be perpendicular to the ground while her right leg should be slightly apart. In this position, she can watch a video she likes placed above her line of sight. To allow for better posture while holding this position, she should wear the brace on her left arm to limit manual stereotypies and put her right arm inside her trousers with the palm facing the buttock (see Figure 60). Have her hold this position for at least 15 minutes a day.

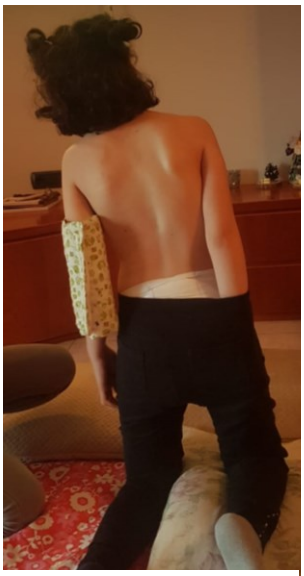

Figure 60

2. Whenever she walks around the house or watches a video while standing, have her wear only her right shoe and a non-slip sock on her left foot. While walking in this way, have her also wear the brace on her left elbow and try to have her right arm inside her pants with the palm facing her buttock (see Figure 61). Try to make it a habit to take off only one of her shoes as soon as she gets home to make sure she walks like this every day for several minutes.

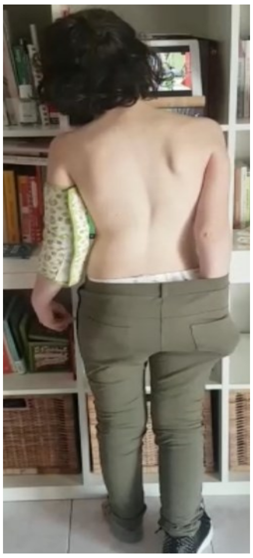

Figure 61

3. One position where she can rest or watch cartoons for long periods is lying on her back with her arms on the sofa's armrest. When you position her in this way, make sure that she is leaning only the arms and eventually the chin on the armrest while the trunk must remain in the air to allow the chest muscles to stretch. This position must be maintained for at least 20 consecutive minutes (if they will be more, even better!) to allow for muscle stretching. In the beginning, to help her get used to the position, you can add a pillow under her chest to reduce the stretch induced by the position (see Figure 62). There are no side effects so that it can be kept as long as possible and used in her daily routine.

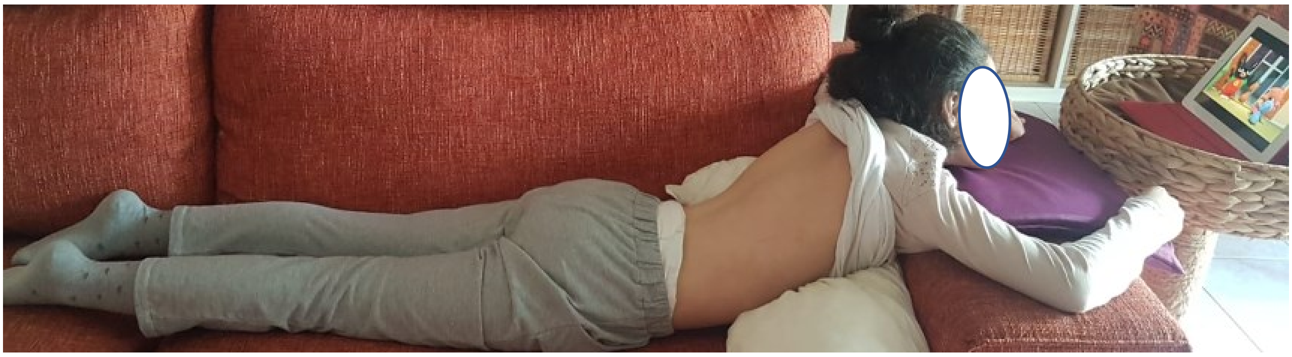

Figure 62

4. Another position she can use to relax is sitting on the floor beside the sofa with her left arm and elbow leaning on the sofa mattress and the right arm inserted in her pants (see image 63). Talk to her while keeping this position to help her tolerate it. Make her holding this position for at least 20 minutes to allow muscles to stretch.

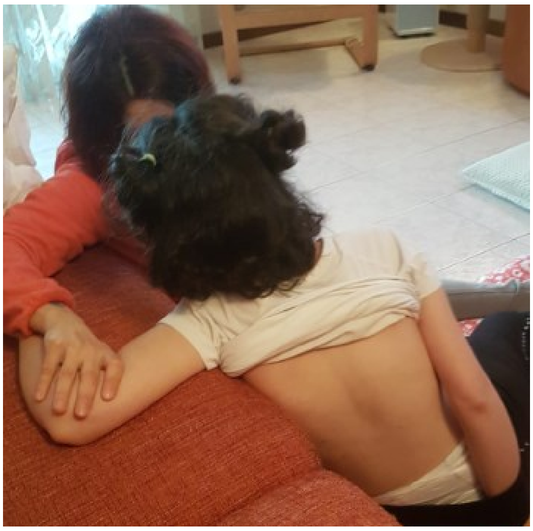

Figure 63

5. She should practice going up and downstairs. To ensure safety, the need for two people to practice the stairs was agreed with the family: one behind and one in front of her to help her in the manner described below.

   a. Climb up - To encourage her to go up, always provide her with a reason to go up the stairs (e.g., go to the bathroom, have a snack, see a video, find another guest in the center, get something, etc.). The person in front of her should keep her hand on the handrail. To help her take the step, move her hand forward on the handrail to unbalance her slightly ahead. Give her clear and simple directions on what she needs to do to climb the step (e.g., “Raise your leg!”; “Push on the leg!”; “Go up!”; Etc.). Wait for her to carry out the movements as autonomously as possible. The person behind her should not give her any support but be there if she lurches back (see Figure 64).

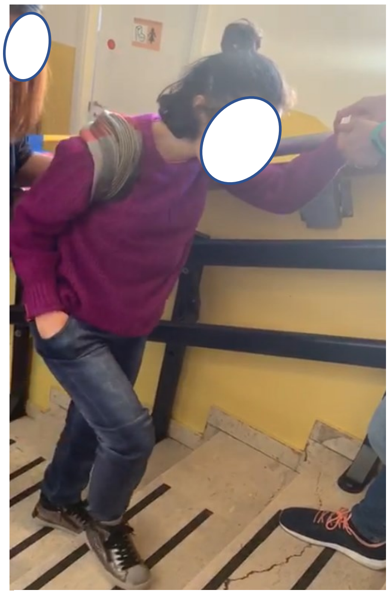

Figure 64

b. Descent - The same method described for going upstairs can also be used to go downstairs. A safety belt can be placed behind her back and under her armpits and held by the operator behind her (see Figure 65) to obtain greater safety. This belt should not offer you any support but should only be used in case of need and potential falls. Remember to give her many compliments every time she goes up or down a step and, in general, every time she carries out a movement independently. Try to do at least 2 flights of stairs a day, both going up and descending.

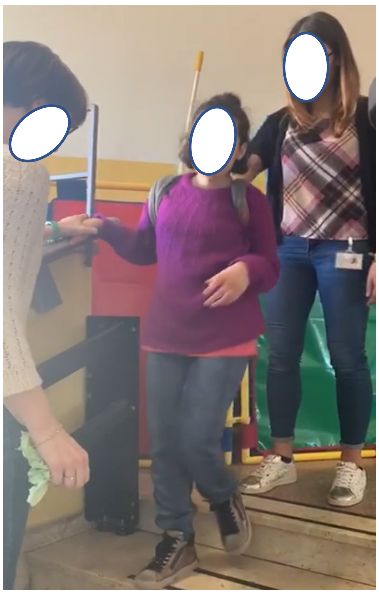

Figure 65

Participant no. 16

1. Every morning, take the time to dance with her 2 or 3 songs she likes before starting the day. Begin these dances with her facing you and holding her by the wrists or pelvis. Start with small, slow movements and gradually increase them in amplitude and speed (see Figure 66). Always try to follow the rhythm of the music. These must be pleasant moments for her. You can do all kinds of movements together with her shoulders, arms, or trunk. When she is set (for example, after the first song), if she is not afraid, you can try to hold her from behind her while facing the television and continue dancing together by moving her body to the rhythm. If there are dancers who make simple movements in the song's video, you can try to make her do them too. Complimenting her when she is having fun. Creating a daily routine in which you dance together by moving her limbs and torso in all directions is also helpful in preventing musculoskeletal abnormalities.

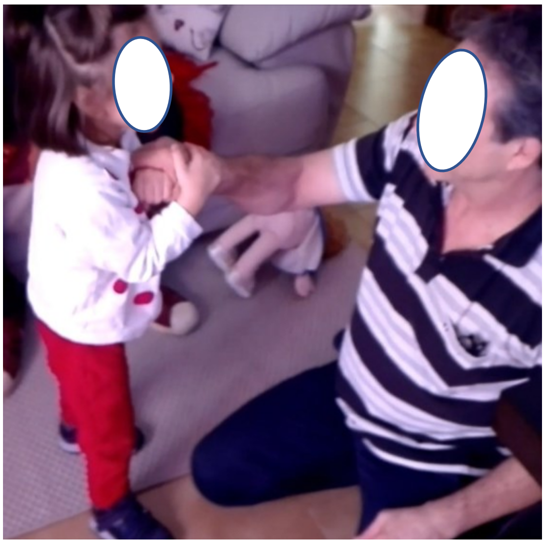

Figure 66

2. Have her sit cross-legged inside the balance plate. Place several cause-and-effect games that she likes around her, so she is motivated to reach them with her hands (see Figure 67). Each time she moves an arm to reach for a game, the plate will cause her to unbalance, and she will have to use her muscles to keep herself seated. For safety, you can add cushions to the side and behind her inside the plate. Have her sit inside the plate while she plays or listen to music on television for at least 20 minutes a day (if they are more, even better!).

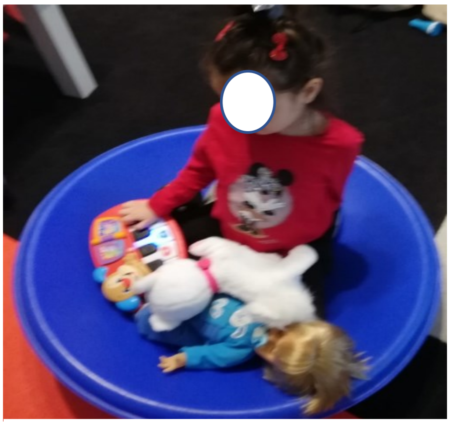

Figure 67

3. She is terrified of walking alone, but, thanks to the fear, if left alone, she can take a few steps independently up to the first available support. She should experience autonomous walking as much as possible to gain confidence. Make sure you create for her at least 10 occasions a day (between home and school) in which you ask her to take a few steps alone. To do this, position yourself behind her a few steps (Initially 3 or 4) far from support (e.g., sofa) or a person. Holding her from her pelvis and, without letting her grab you with her hands, wait for her to unbalance towards the support or the other person slightly, then, slowly, release her and let her take the few steps that separate her from the support (see Figure 68). Try to do this activity every day.

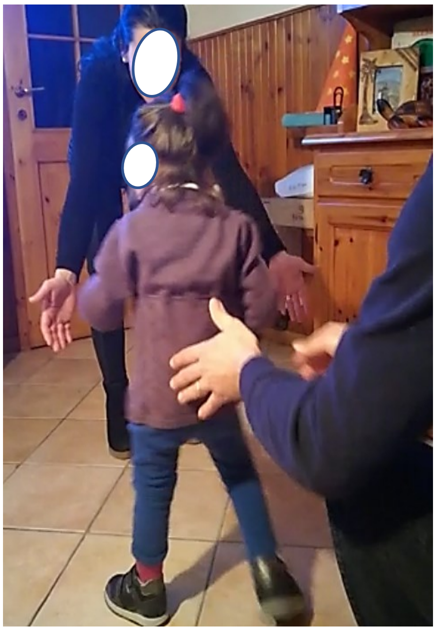

Figure 68

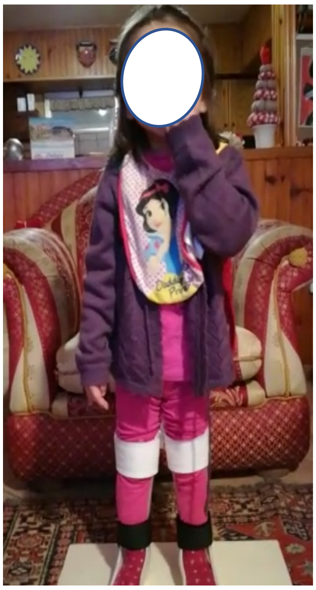

Figure 69

4. Have her stand inside the standing frame built by screwing two AFO splints to a wooden board while looking at the cartoons (see Figure 69). While she is standing in this standing frame, you can also dance with her to the music as proposed in activity 1. Make her stand in this way without support to the hands as much as possible. Try to reach at least 20 minutes a day.

Participant no. 17

1. Have her sit without back support on the wedge pillow oriented diagonally forward and to the right (her left buttock is higher than the right). While she is in this position, the adult should stand to her left and hold her left knee with his leg (see Figure 70). When she begins to get tired, she can rest for 1 or 2 minutes by leaning on her left hand before continuing to hold the position for a few more minutes. She should sit like this for as long as possible but try to make her reach at least 20 minutes a day. To achieve the complete duration of the exercise, start with short sessions at different times of the day (e.g., 4 sessions of 5 minutes) and progressively lengthen the duration of the sessions.

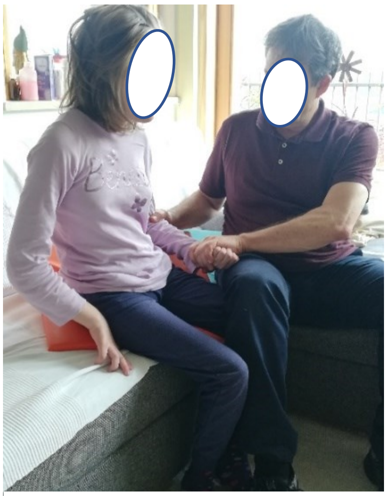

Figure 70

2. Have her stand leaning against a wall or piece of furniture behind her while watching a video placed to her right (see Figure 71). Always use the AFO splint for this activity. The first few times she tries, she could need help to avoid turning her pelvis to the right and leaning forward. Try to keep her in this position as well for at least 20 minutes a day. Start with shorter durations and progressively increase the exercise times.

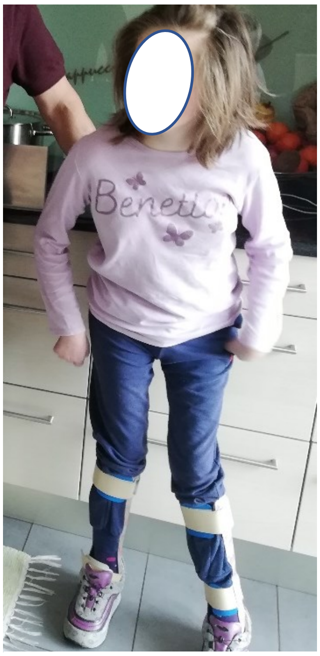

Figure 71

3. Have her walk as much as possible throughout the day, independently with her walker or on the treadmill with support from behind (see Figures 72a, b). As she walks, always have her load the weight on her legs and take the step alone. To ensure that she walks enough every day, you can identify some activities of her day to reach by walking.

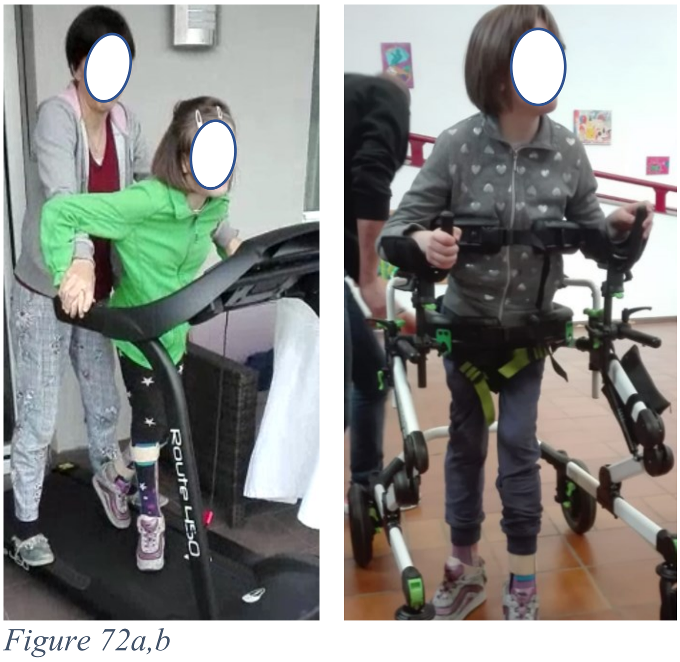

Figure 72a,b

4. To prevent the progression of scoliosis and allow the muscles of the left side of the trunk to stretch, she should rest lying on her right side inside the "U" shaped pillow. The thickness of the pillow should be placed below the apex of her scoliotic curve (see Figure 73). This position should be maintained without a pillow under the head. Initially, to get it used to, you can use a low cushion under the head and gradually reduce its thickness until it is completely removed. This position should be maintained for at least 20 consecutive minutes to allow for muscle stretching.

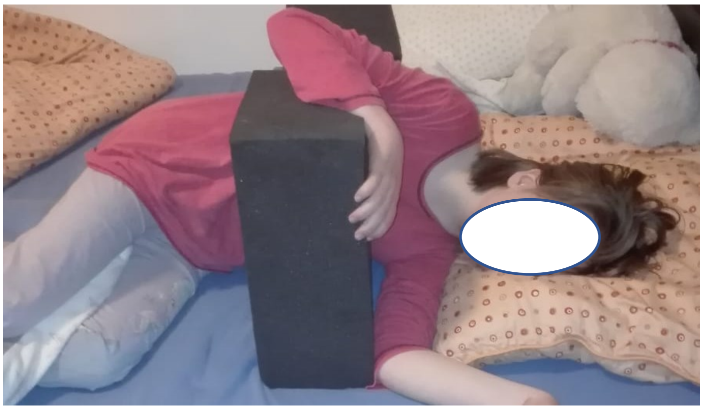

Figure 73

5. To extend the anterior muscle chain of the trunk, she should lie prone with her arms on the armrest of the sofa. Ensure that she is leaning only her arms and elbows on the armrest and not the trunk to encourage the spine extension (see Figure 74). Have her maintaining this position for at least 20 consecutive minutes to allow muscle stretch. This position and the one described in activity 4 have no side effects and can be kept as long as possible and used in her daily routine whenever she needs to rest.

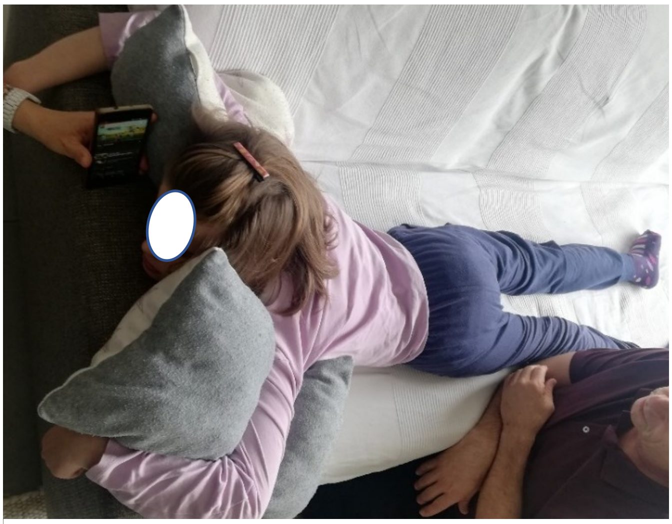

Figure 74

Participant no. 18

1. Have her sit on the edge of a stool with her right hip extended and her right knee resting on the ground next to the stool (see Figure 75). The person doing this exercise with her should sit in front of her to allow her to lean on his shoulder, helping her hold the position. This exercise activates the muscles of the left side of the trunk and exercises them by counteracting the progression of scoliosis. Have her hold this position every day initially for 2 sessions of a few minutes (5-10) and gradually increasing the exercise duration until you perform at least one 20-minute session a day.

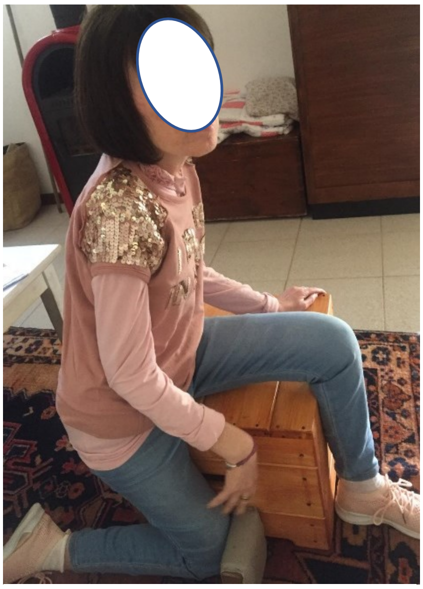

Figure 75

2. Have her sit on the sofa and bend her right leg so that her right foot is behind her left knee. Sit in front of her while she holds this position and hold her right hand with yours to facilitate the rotation of the trunk to the left (see Figure 76). In addition, to promote the extra rotation of the right hip, you can put a weighted bag (e.g., full of sand) on her right knee to keep it close to the sofa mattress. Alternatively, you can use your left hand. Try to have her hold this position for at least 20 minutes a day, starting with several sessions of a few minutes each.

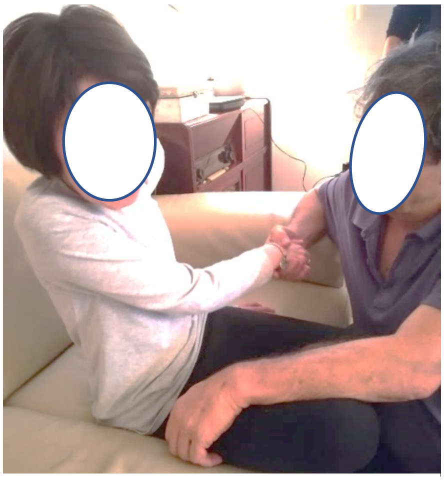

Figure 76

3. When she is tired and needs to rest, try letting her relax in a prone position on the sofa while keeping her elbows slightly forward of her shoulders to allow for the stretching of the anterior muscle chain (see Figure 77). Have her hold this position for at least 20 continuous minutes to allow the muscles to relax and stretch (if they are more, even better!).

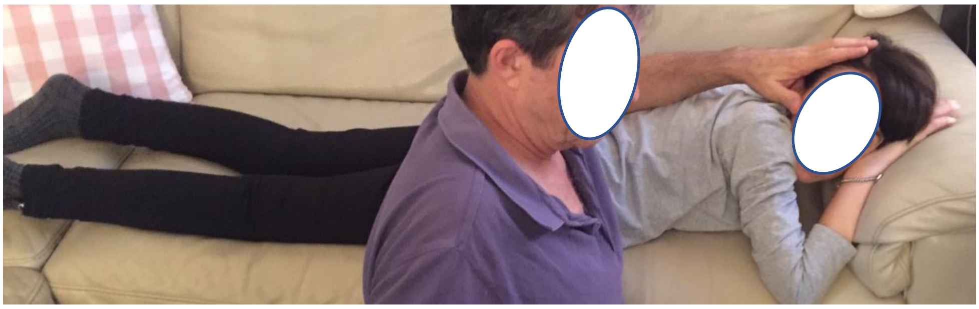

Figure 77

4. Spend at least 20 continuous minutes a day sitting with her while keeping her arms around your shoulders (see Figure 78). This will improve the mobility of her shoulders and her spine. To make this moment pleasant for her, you can tell each other about the day's episodes or watch a movie together while holding the position.

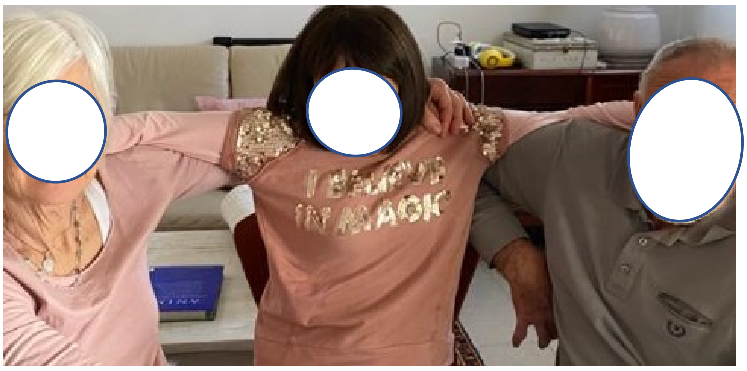

Figure 78

Participant no. 19

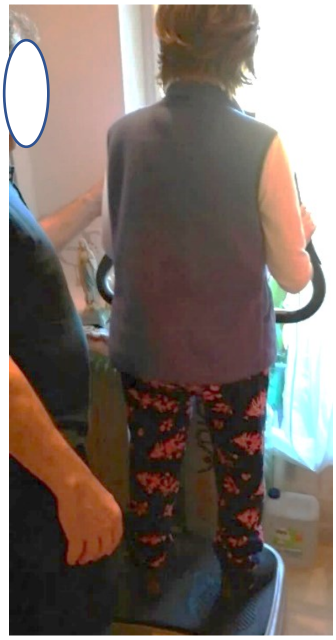

Figure 79

1. Her exercises require important muscular activity. To stimulate the activation of her muscles, have her perform a 10-minute session on the vibrating platform at a frequency of 40 hertz and with movements of 0.2 mm in amplitude before starting the exercises (see Figure 79).

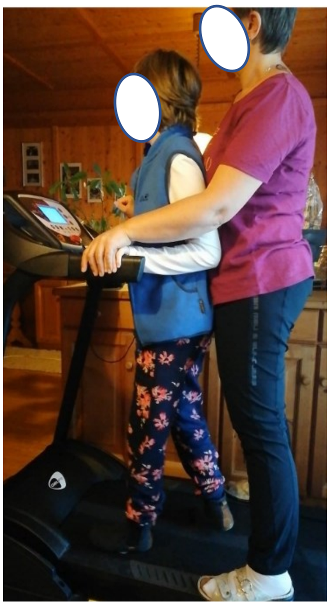

Figure 80

2. Have her walk on the treadmill by positioning you behind her (see Figure 80) every day at 2 km/h and with a 10° incline for 10 minutes while she looks at her favorite cartoon. Monitor her heart rate and if it exceeds 140 BPM, have her stop for a few minutes of rest before resuming. Initially, you can split the 10-minute session into two 5-minute sessions separated by a one-minute break.

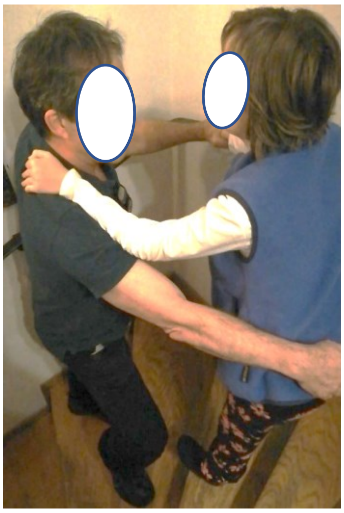

Figure 81

3. Have her go down a flight of stairs at least twice a day by standing in front of her. Place her hand against your shoulder and keep her other hand on the handrail. Use your free hand to help her keep balance (see Figure 81). Always give her a reason to go down the stairs (e.g., let's visit grandma, go for a snack, etc.) Throughout the descent of the stairs, guide each movement with a precise verbal indication (e.g., if she has to bend the knee, say: "Bend the knee!") And give her time to organize and carry out the movement. At first, it may take her some time to get used to going down with one hand on the handrail. And the end of the stairs, have her doing the last 2 or 3 steps supporting her only by one wrist and with the other hand on the handrail (to exercise the descent to a greater level of autonomy).

4. Have her go up a flight of stairs at least twice a day. To help her, position yourself in front of her and keep her wrist firmly and her other hand firmly on her handrail. Use elastic bandages to help her move her feet to the next step with the movement of your legs (see Figure 82). Always give her simple and precise verbal directions on what to do (e.g., "Raise your foot!", "Push on the leg!", etc.). Let her hold her weight and give her enough time to do the movement. Remember to move the hand on the handrail forward after each step.

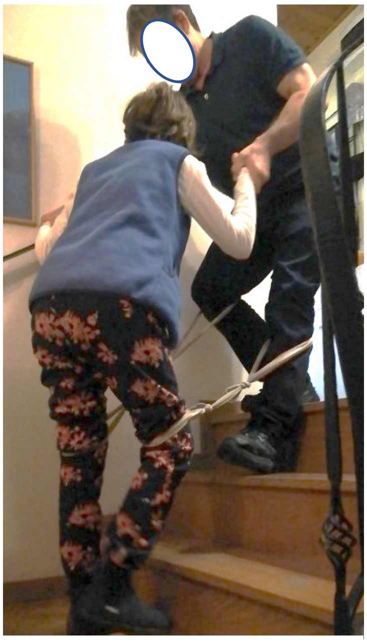

Figure 82

5. Build an obstacle course for her with different heavy objects and materials of different textures arranged on the floor of the living room (e.g., chairs, rugs with hard or soft small and medium-sized objects underneath, cushions, etc.) and ask her to walk across the room alone to reach something of interest (e.g., a family member, a video, some food, etc.) (see Figure 83). Have her walk inside the obstacle course for at least 20 minutes a day.

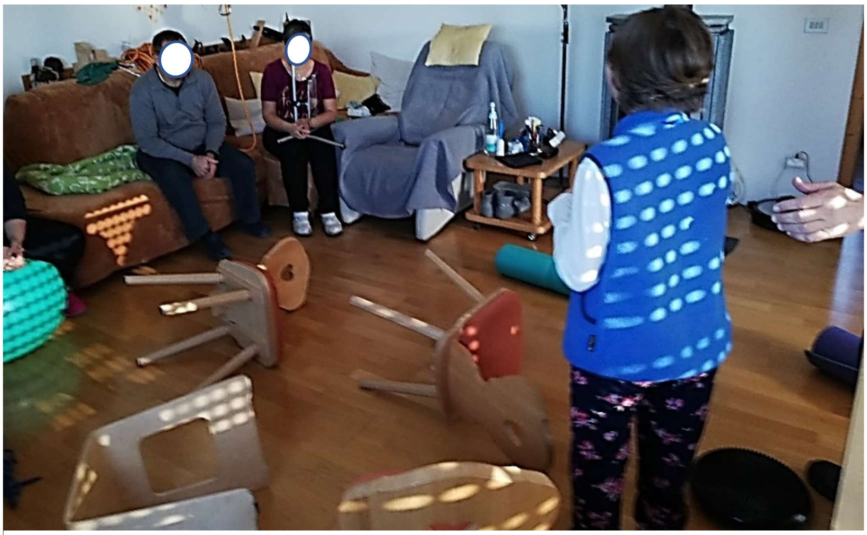

Figure 83

6. Have her sit for 20 consecutive minutes a day on the back half of the inflatable pillow (further back than she is sitting in Figure 84) with no back or foot support to encourage the use of the abdominal muscles and pelvic anteversion. Have her look at something interesting placed at her eye level while she holds this position.

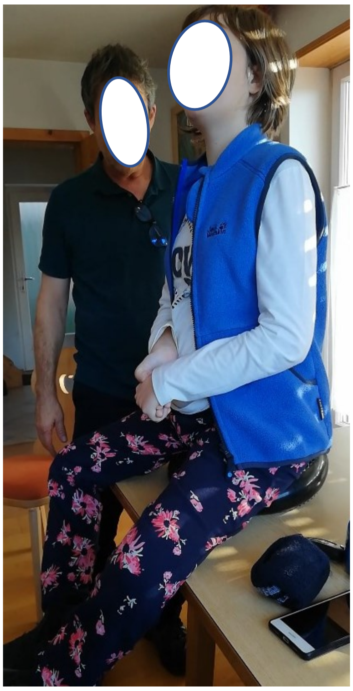

Figure 84

Participant no. 20

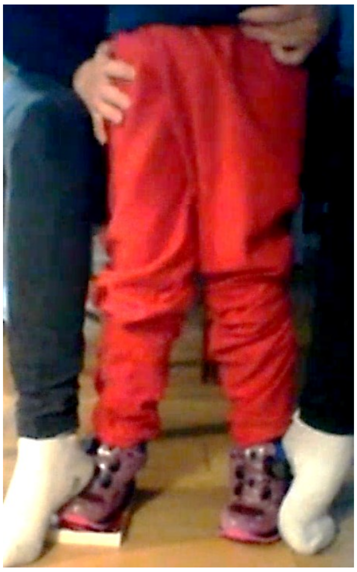

Figure 85

1. Make her stand up by holding her by the pelvis while keeping her right foot on a rise of at least 3cm (e.g., a book or a tile) to unbalance her to her left and activating her balancing reaction against her scoliotic curve (see figure 85). Have her hold this position for at least 10 minutes a day. Especially initially, if she cannot control the position for the entire exercise duration, start with shorter sessions (e.g., 2 sessions of 5 minutes) and gradually increase their duration.

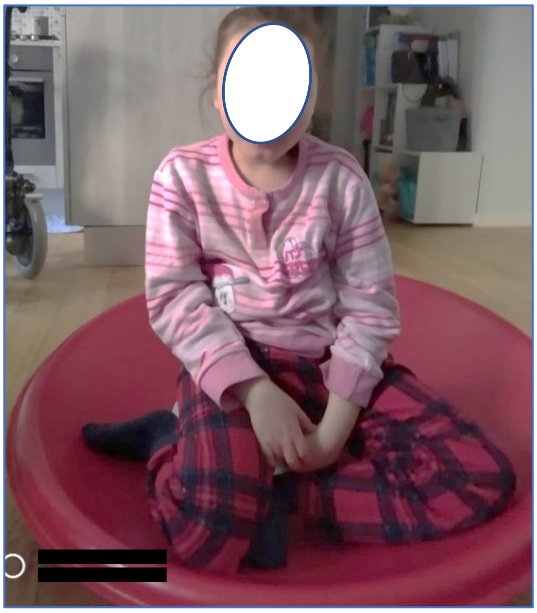

Figure 86

2. Have her sit alone on a stool positioned inside the balance plate with her knees pointing to the left to stimulate the activation of the right-side torso musculature (see Figure 86) while she is looking at something of interest placed in front of her. Ensure she cannot put her hands together while sitting like this and pay attention to the pelvis position as it tends to slide forward, assuming an incorrect posture. Have her hold this position for at least 15 minutes. Start with several sessions of shorter duration and gradually increase the times until you reach the complete duration of the exercise.

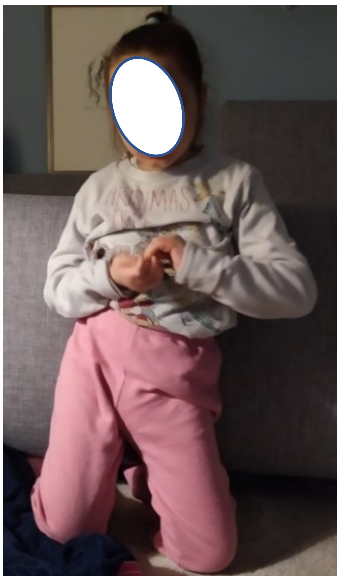

Figure 87

3. Have her kneeling with the hips extended and a slight rise below the right knee (about 3 cm, e.g., a folded beach towel) to strengthen the musculature of the right side of her trunk and counteract scoliosis. Next, place a sofa cushion on her calves behind her back have her leaning on it. While holding this position, her left leg should be well perpendicular to the ground while her right leg should be slightly apart (see Figure 87). In this position, she can watch a video she likes that is placed slightly above her line of sight. Have her hold this position for at least 15 minutes a day. Also, for this position, start with several sessions of fewer minutes and gradually increase their duration and reduce their number (e.g., first 3 sessions of 5 minutes, then 2 sessions of 8 minutes) until you reach the entire length of the exercise.

4. Have her sit on the stool on the floor in front of the sofa with her knees pointing to the left and her left elbow leaning on the sofa's mattress (see Figure 88). Compared to how it is positioned in Figure 95, the stool should be slightly further away from the sofa (see red arrow) so that the right side of its trunk results more extended. Place interesting toys to her right (e.g., soft toys, light, and sound games, videos) so that she has an incentive to turn to the right to look at them. To keep her left arm in place, you can add a scarf wrapped around her arm and tuck it into the sofa cushions. Pay attention to the trunk position because it tends to slide forward, assuming an incorrect posture. Have her hold this position for at least 20 consecutive minutes. This position can also be held unsupervised, eventually adding some cushions around her for safety.

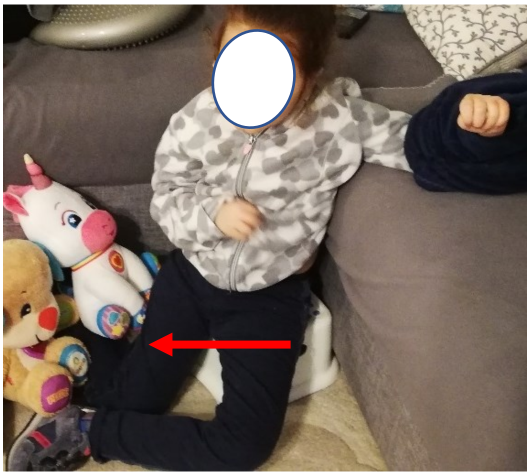

Figure 88

5. When she needs to rest, she can do it lying on her back on the sofa with a rolled-up towel under her chest (placed just below her armpits) (see Figure 89). This position has no side effects and can be maintained even for long periods without supervision, counteracting kyphosis and stretching the anterior trunk muscles. However, to allow the muscles to relax and stretch, this position must be held for at least 20 consecutive minutes.

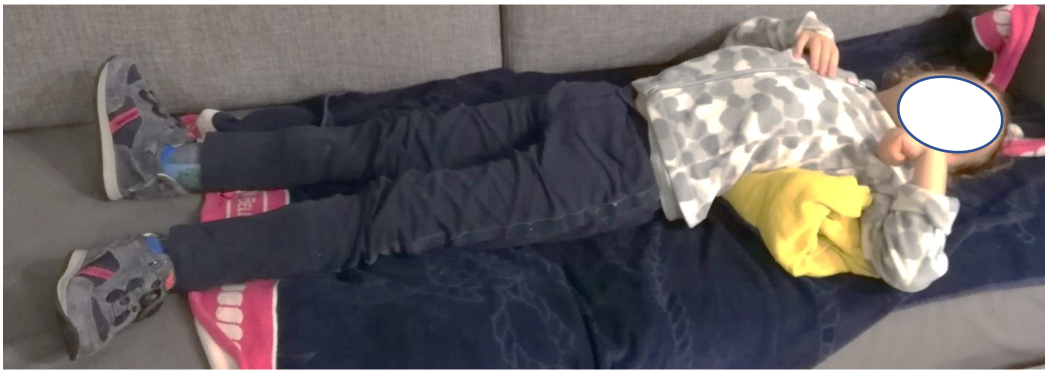

Figure 89

6. Another position for her to rest is lying prone on the ground with her arms leaning on a roll (e.g., made out of towels - about 20cm in diameter). Ensure that only her arms leaning on the roll and not her chest (to allow her chest muscles to stretch). In this position, bend her right knee and hip to encourage elongation of the trunk's left side, counteracting scoliosis (see Figure 90). This position should also be held for at least 20 consecutive minutes to be effective.

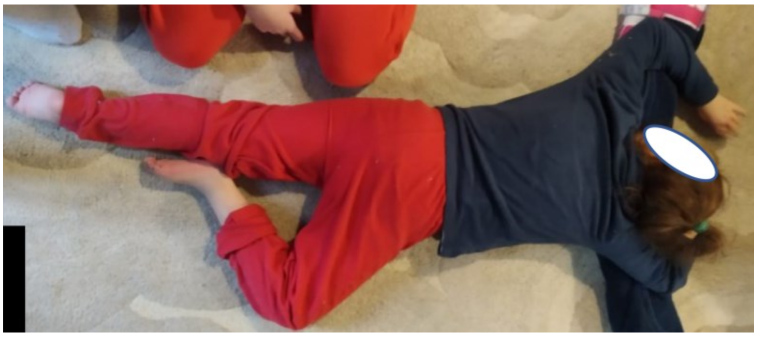

Figure 90
