# Supplementary material for: Intensive Postural and Motor Activity Program Reduces Scoliosis Progression in People with Rett Syndrome
Source: J Clin Med. 2022 Jan 22;11(3):559. doi: 10.3390/jcm11030559 (PMC8837095; doi:10.3390/jcm11030559)
Supplement: Supplementary file 1 [file jcm-11-00559-s001.zip › Tables ¿C Supplementary material.pdf]

Table S1. Individual data of each participant's age, genetic mutation, RARS subscales and total scores, and daily physical activity level at T1.

No.	Mutation	Age at T1	RARS at T1							m-BAR at T1
			Cognitive	Sensory	Motor	Emotional	Indipendence	Rett characteristics	Total	
1	R306C	11,5	12,5	4	13	4	11,5	29,5	74,5	74
2	P152R	22,0	12,5	3	9,5	2,5	9,5	24,5	61,5	101
3	pro225arg	27,7	15,5	5,5	9,5	4	11	22	67,5	62
4	c.468 C>G	13,1	15	3	13	3	12	25	71	52
5	R168*	17,2	10,5	2	5,5	2,5	10	19,5	50	121
6	R306C	12,8	18,5	5	10,5	5	11	27	77	130
7	R133C	12,3	13,5	2,5	7	3	4,5	15	45,5	97
8	T158M	6,5	17,5	4	9	4	11,5	19	65	124
9	P133C	13,3	15,5	3	10,5	4	10,5	29,5	73	81
10	R255*	10,3	15	2	8,5	3	12	21,5	62	102
11	R255*	31,6	10,5	3,5	12,5	3,5	12	25	67	65
12	T158M	16,7	11,5	2	13	3	11	25,5	66	73
13	R106W	14,4	13,5	4	13	3	11,5	21	66	90
14	T158M	12,7	10,5	3	10	2,5	9	19,5	54,5	109
15	c. 1063_1326del264 + c. 1063insTGCG	13,6	11	2	10,5	2	12	24	61,5	105
16	R294X	3,8	14	5	10	2,5	10	27,5	69	145
17	R294X	12,9	16,5	3	13	4	12	33	81,5	56
18	T158M	38,3	9,5	2	8,5	3	9	23,5	55,5	110
19	R255X	13,3	16	3,5	11	4	12	27	73,5	89
20	R168X	8,8	9	2	9,5	2	12	17	51,5	69

Table S2. Individual data of each participant's RESMES subscales and total scores measured at T1 and T2.

No.	RESMES at T1							RESMES at T2						
	**Standing**	**Sitting**	**Transition**	**Walking**	**Run**	**Stairs**	**Total**	**Standing**	**Sitting**	**Transition**	**Walking**	**Run**	**Stairs**	**Total**
1	4	9	18	14	4	8	57	7	3	17	17	4	8	56
2	1	0	16	6	4	6	33	0	0	14	4	4	6	28
3	2	0	18	7	4	6	37	0	0	17	6	4	6	33
4	6	1	24	17	4	8	60	7	0	24	14	4	8	57
5	0	0	13	2	4	6	25	0	0	13	1	4	6	24
6	0	0	7	0	4	4	15	0	0	7	0	4	4	15
7	0	0	0	0	4	2	6	0	0	0	0	4	2	6
8	1	0	20	2	4	8	35	0	0	20	2	4	6	32
9	0	0	13	5	4	7	29	0	0	13	3	4	6	26
10	0	0	16	5	4	6	31	0	0	13	5	4	6	28
11	11	0	22	18	4	8	63	8	0	21	16	4	8	57
12	11	5	24	18	4	8	70	10	2	24	18	4	8	66
13	0	0	8	2	4	6	20	0	0	7	3	4	6	20
14	6	3	19	15	4	8	55	6	3	19	14	4	8	54
15	0	0	7	0	4	6	17	0	0	7	0	4	4	15
16	4	0	10	6	4	8	32	3	0	10	5	4	7	29
17	8	1	21	15	4	8	57	4	0	22	12	4	8	50
18	0	0	7	2	4	3	16	0	0	7	2	4	3	16
19	0	0	9	0	4	6	19	0	0	9	1	4	4	18
20	9	0	19	18	4	8	58	7	0	18	18	4	8	55

Table S3. Individual data of each participant's scoliosis severity level at T1, time elapsed between pre- (T1) and post- (T2) intervention spine radiographs, Cobb’s angles measured at T1 and T2, and Cobb’s angle variation between T1 and T2. Participants’ scoliosis severity level was coded with a 0 to 3 ordinal score (0: no scoliosis; 1: mild scoliosis, curve visible only on thorough examination in forwarding bending; 2: moderate scoliosis, obvious curve in both upright and forward bending; 3: severe scoliosis, pronounced curve preventing upright position without external support).

**N°**	**Scoliosis severity at T1**	**Months between T1-T2**	**Cobb's angle at T1 (°)**	**Cobb's angle at T2 (°)**	**Curve convexity**	**Δ Cobb's angle (°)**
1	2	7,9	23	24	Rt	1
2	2	10,2	33	28	Rt	-5
3	2	15,3	32	36	Rt	4
4	3	12,5	27	41	Lt	14
5	1	14,0	11	24	Lt	12
6	1	17,7	14	18	Lt	5
7	2	16,6	20	27	Rt	7
8	1	14,6	10	0	Lt	-10
9	2	18,4	25	21	Lt	-4
10	2	9,8	21	19	Rt	-2
11	3	8,9	41	30	Rt	-11
12	3	8,4	16	17	Rt	2
13	3	9,3	42	65	Lt	23
14	3	10,0	56	58	Rt	2
15	2	11,8	24	30	Rt	6
16	1	11,8	13	1	Rt	-13
17	3	7,4	39	36	Lt	-3
18	3	17,3	41	39	Lt	-3
19	1	10,3	13	16	Rt	4
20	3	13,4	8	14	Rt	6
